# Supplementary material for: 11C-radiolabeled aptamer for imaging of tumors and metastases using positron emission tomography- computed tomography
Source: Mol Ther Nucleic Acids. 2021 Oct 21;26:1159–72. doi: 10.1016/j.omtn.2021.10.020 (PMC8601970; doi:10.1016/j.omtn.2021.10.020)
Supplement: Document S1. Figures S1–S5 [file mmc1.pdf]

OMTN, Volume *26*

Supplemental information

^{11}C-radiolabeled aptamer for imaging of tumors and metastases using positron emission tomography- computed tomography

Anastasia V. Ozerskaya, Tatiana N. Zamay, Olga S. Kolovskaya, Nikolay A. Tokarev, Kirill V. Belugin, Natalia G. Chanchikova, Oleg N. Badmaev, Galina S. Zamay, Irina A. Shchugoreva, Roman V. Moryachkov, Vladimir N. Zabluda, Vladimir A. Khorzhevskii, Nikolay Shepelevich, Stanislav V. Gappoev, Elena A. Karlova, Anastasia S. Saveleva, Alexander A. Volzhentsev, Anna N. Blagodatova, Kirill A. Lukyanenko, Dmitry V. Veprintsev, Tatyana E. Smolyarova, Felix N. Tomilin, Sergey S. Zamay, Vladimir N. Silnikov, Maxim V. Berezovski, and Anna S. Kichkailo

^{11}C-Radiolabeled Aptamer For Imaging of Tumor and Metastasis Using Positron Emission Tomography - Computed Tomography

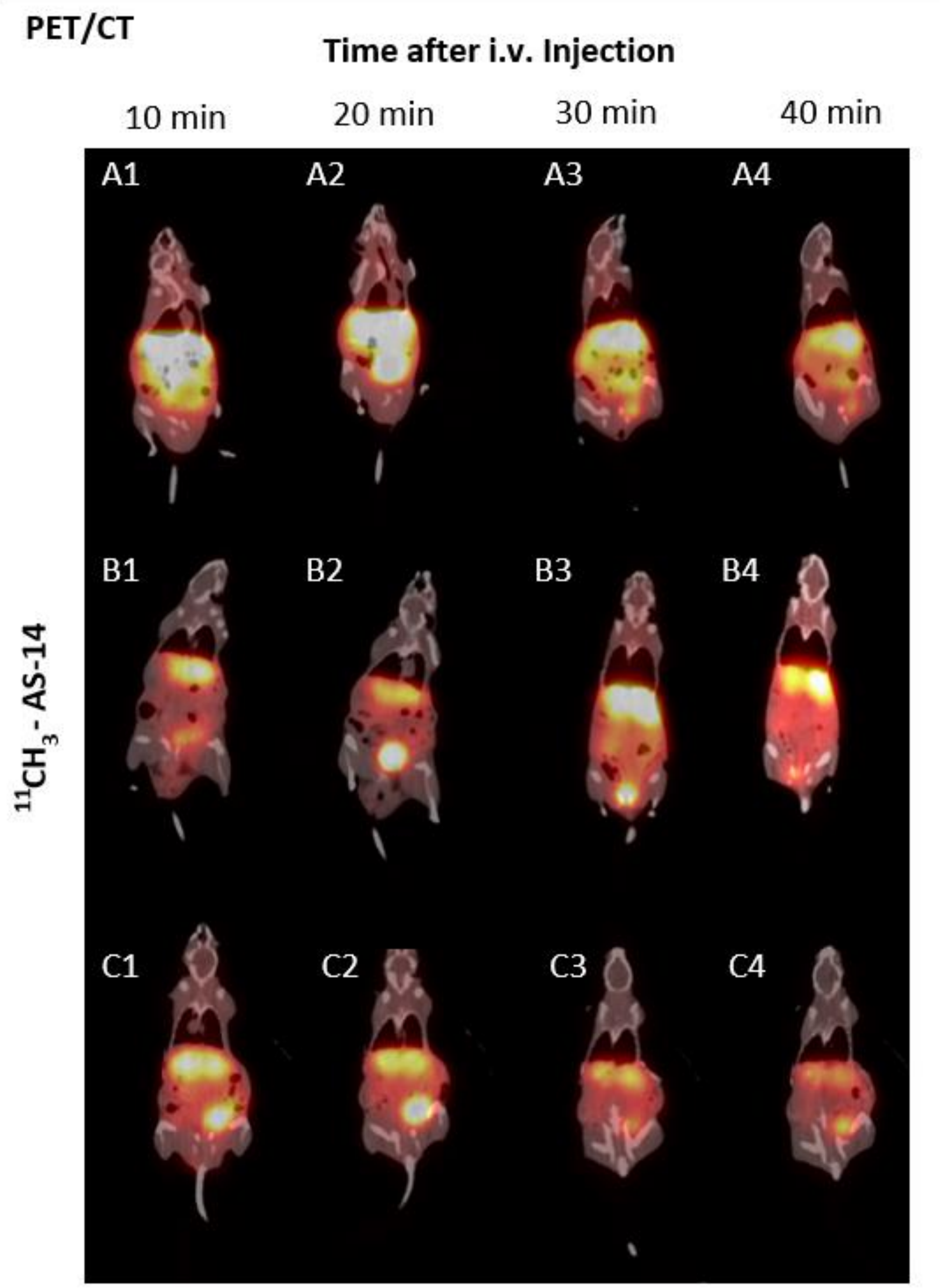

Figure S1. PET/CT imaging of $^{11}CH_3$-AS-14 of three mice with Ehrlich's tumor metastases. The scans were performed at 10, 20, 30, and 40 minutes after tail vein injection of 0.4 nmol $^{11}CH_3$-AS-14 in 200 µl of DPBS.

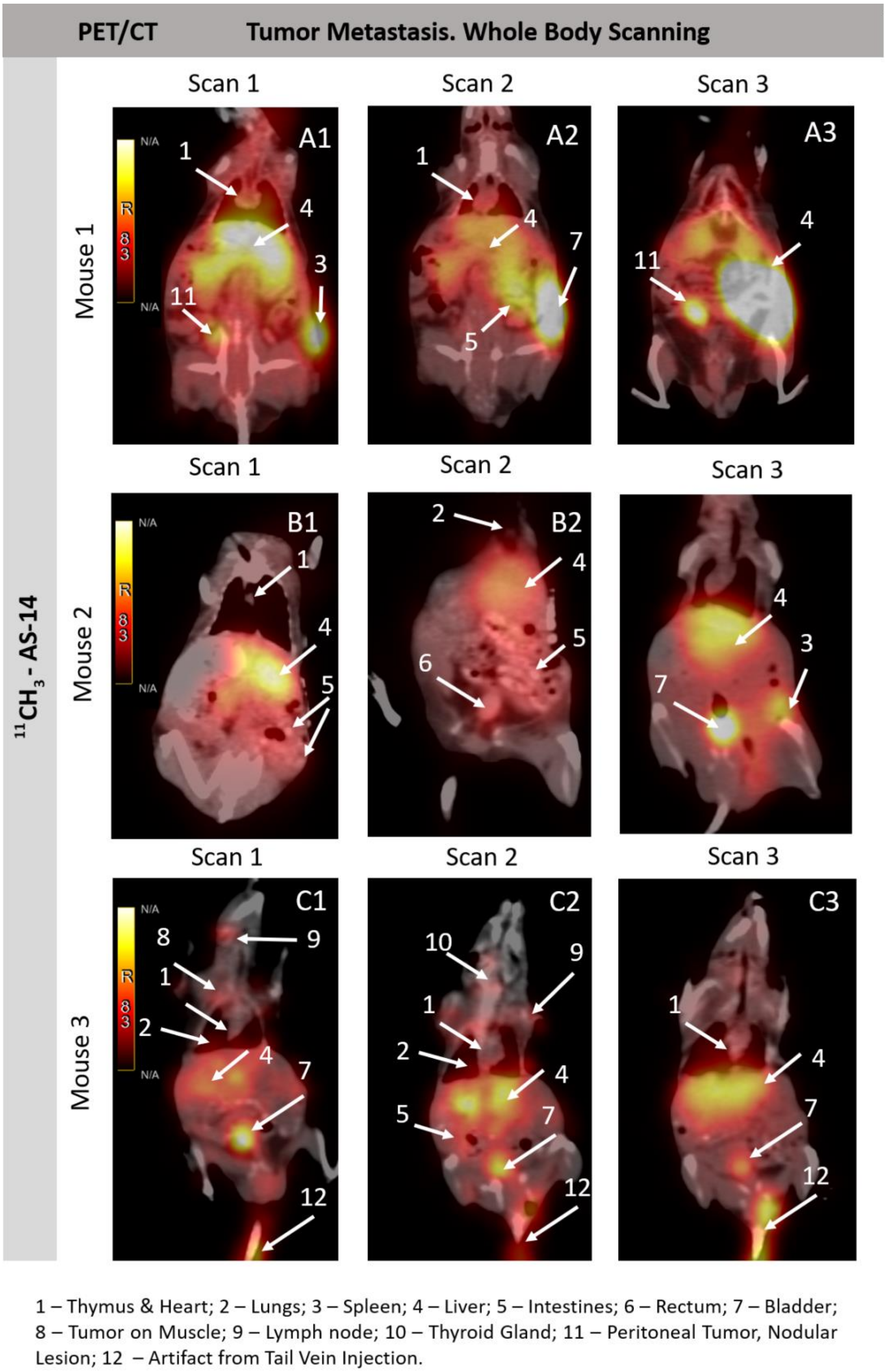

Figure S2. Whole body PET/CT scanning after injection of $^{11}CH_3$-AS-14 probe reveals tumor localization in different organs. The PET/CT images of 3 representative mice with metastases (3 scans per mouse). Arrows indicate the tumor sites in different organs.

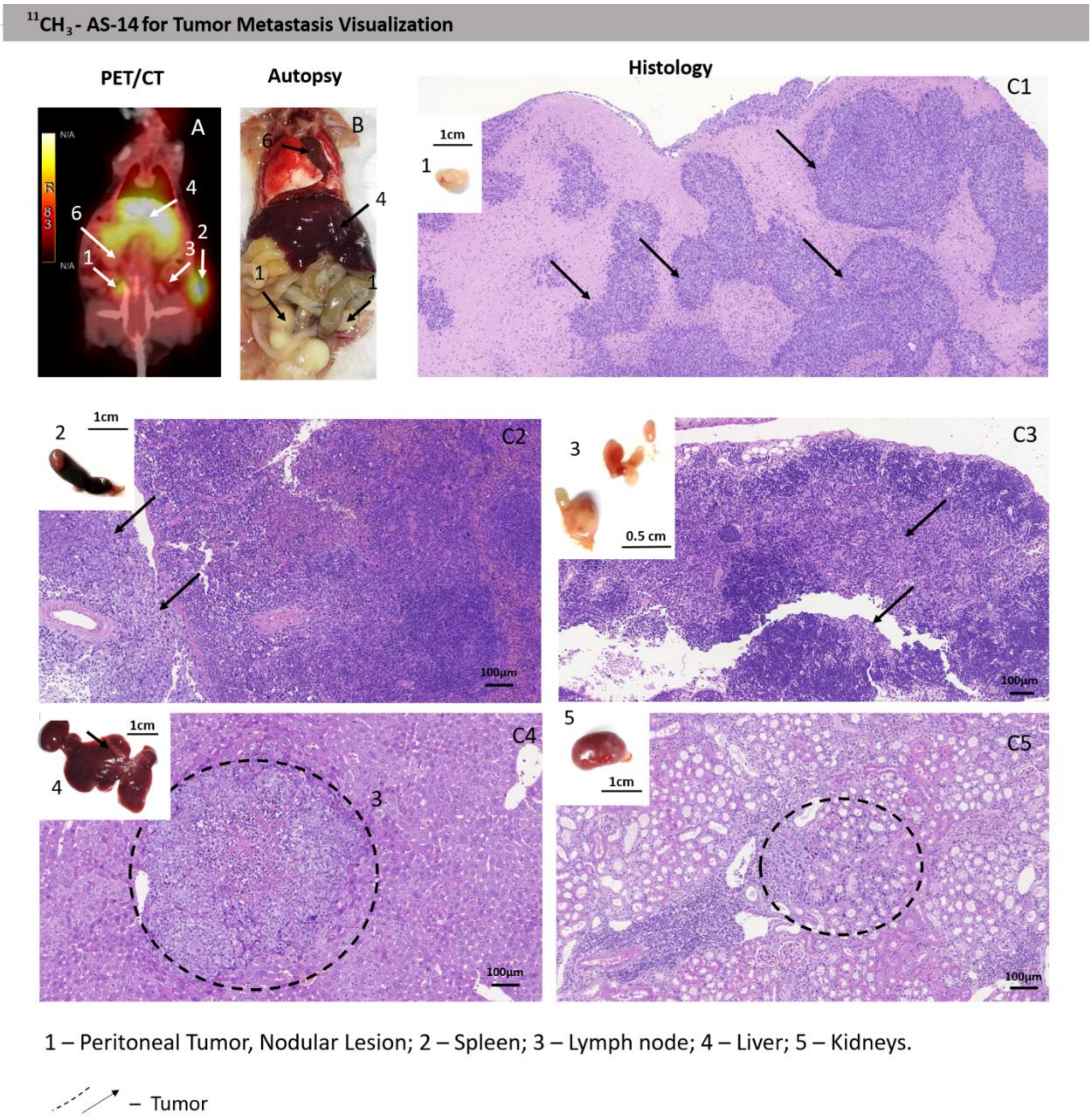

Figure S3. Tumor and metastases localization using the $^{11}CH_3$-AS-14 probe and PET/CT. The representative PET/CT images of mice with metastases (A) were confirmed by autopsy (B) and histological analyses of metastatic tissues (C): peritoneal tumor, nodular lesion (C1), spleen (C2), lymph node (C3), liver (C4), and kidneys (C5). Hematoxylin & Eosin, staining. Magnification C1 – ×30; C2,5 - ×100. Arrows and dashed circles indicate the tumor sites in different organs at PET/CT, autopsy, and correspondent tissue sections.

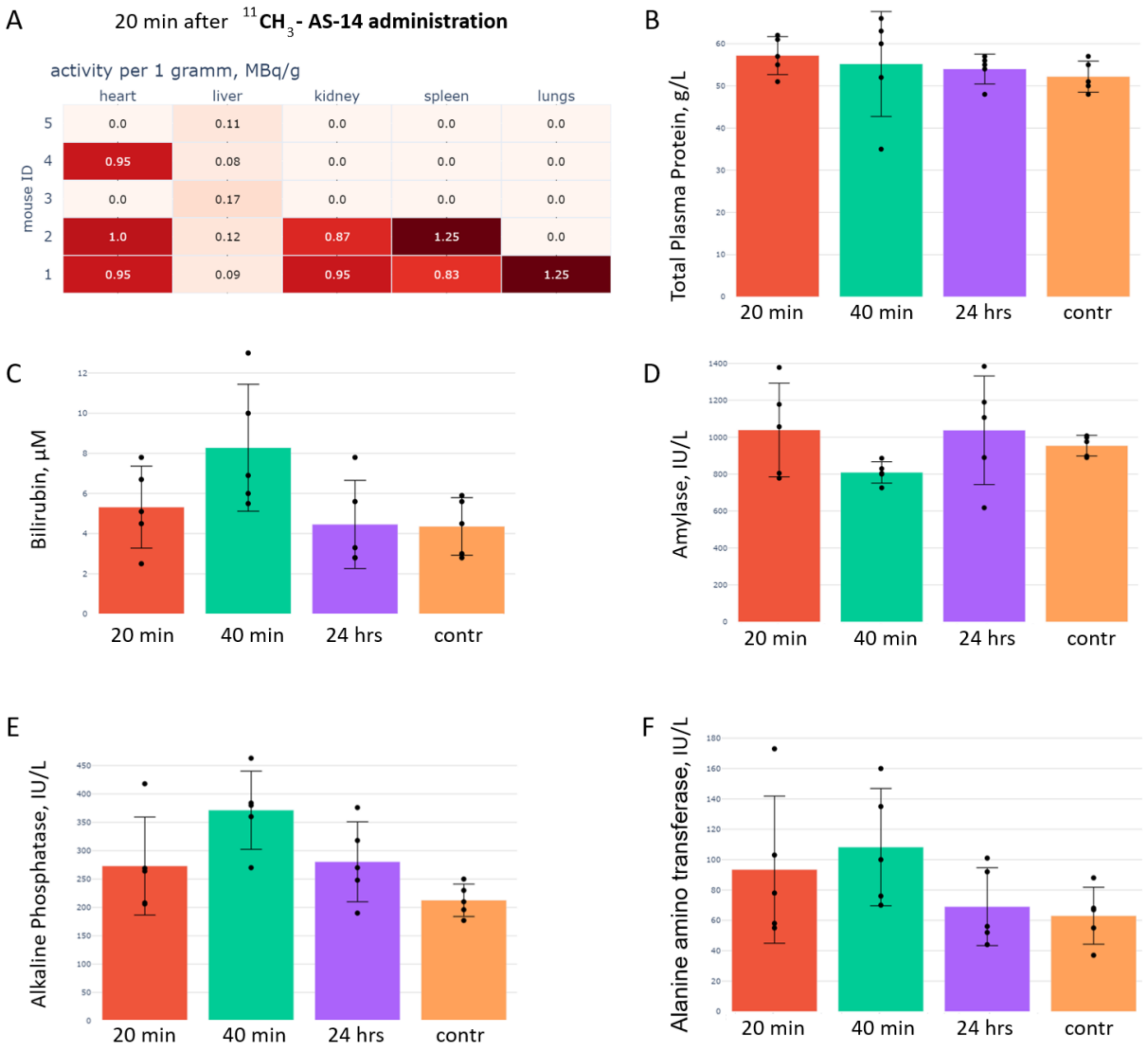

Figure S4. Acute toxicity study of $^{11}CH_3$-AS-14. Accumulation of radioactivity in different mouse organs after 20 minutes of intramuscular $^{11}CH_3$-AS-14 administration (A). Blood serum biochemistry after 20, 40 minutes and 24 hours of intramuscular $^{11}CH_3$-AS-14 administration: levels of total plasma protein (B) and bilirubin (C), activities of amylase (D), alkaline phosphatase (E) and alanine aminotransferase (F).

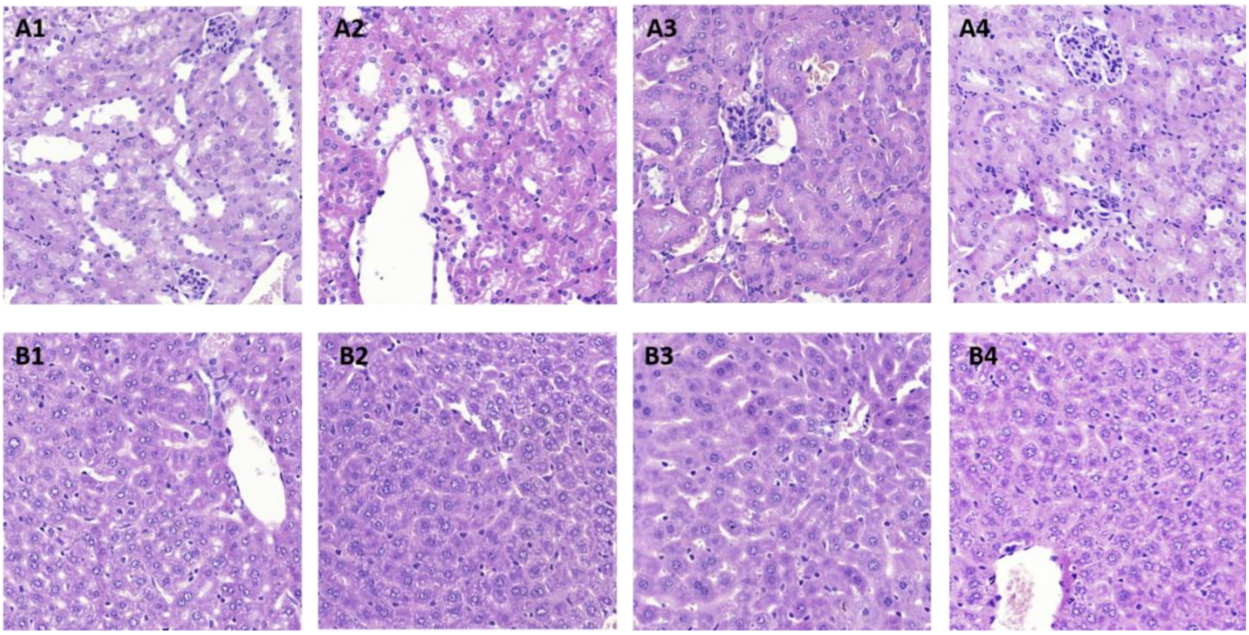

Figure S5. Histological examinations of mice kidneys and livers after intramuscular $^{11}CH_3$-AS-14 administration. Kidneys (A) and livers (B) after 20 (1), 40 (2) minutes and 24 hours (3) comparing with a DPBS-injected control group (4).

Supplemental Video. Comparative PET/CT imaging using $^{11}CH_3$-AS-14 with ^{18}F-FDG.
