# Supplementary material for: 11C-radiolabeled aptamer for imaging of tumors and metastases using positron emission tomography- computed tomography
Source: Mol Ther Nucleic Acids. 2021 Oct 21;26:1159–72. doi: 10.1016/j.omtn.2021.10.020 (PMC8601970; doi:10.1016/j.omtn.2021.10.020)
Supplement: Document S2. Article plus supplemental information [file mmc3.pdf]

Molecular Therapy
Nucleic Acids
Original Article

^{11}C-radiolabeled aptamer for imaging of tumors and metastases using positron emission tomography- computed tomography

Anastasia V. Ozerskaya,[1] Tatiana N. Zamay,[2,3] Olga S. Kolovskaya,[2,3] Nikolay A. Tokarev,[1] Kirill V. Belugin,[1] Natalia G. Chanchikova,[1] Oleg N. Badmaev,[1] Galina S. Zamay,[2,3] Irina A. Shchugoreva,[3] Roman V. Moryachkov,[3,4] Vladimir N. Zabluda,[4] Vladimir A. Khorzhevskii,[2,7] Nikolay Shepelevich,[1] Stanislav V. Gappoev,[2,7] Elena A. Karlova,[1] Anastasia S. Saveleva,[1] Alexander A. Volzhentsev,[1] Anna N. Blagodatova,[2] Kirill A. Lukyanenko,[2,3] Dmitry V. Veprintsev,[3] Tatyana E. Smolyarova,[3,4] Felix N. Tomilin,[4] Sergey S. Zamay,[3] Vladimir N. Silnikov,[5] Maxim V. Berezovski,[6] and Anna S. Kichkailo[2,3]

[1]Federal Siberian Research Clinical Centre Under the Federal Medical Biological Agency, Krasnoyarsk, Russia; [2]Krasnoyarsk State Medical University named after Prof. V.F. Voino-Yasenetsky, Krasnoyarsk, Russia; [3]Federal Research Center Krasnoyarsk Science- Center SB RAS, Krasnoyarsk, Russia; [4]Kirensky Institute of Physics, Krasnoyarsk, Russia; [5]Institute of Chemical Biology and Fundamental Medicine, Siberian Branch of the Russian Academy of Sciences, Novosibirsk, Russia; [6]Department of Chemistry and Biomolecular Sciences, University of Ottawa, Ottawa, Canada; [7]Krasnoyarsk Regional Pathology-Anatomic Bureau, Krasnoyarsk, Russia

Identification of primary tumors and metastasis sites is an essential step in cancer diagnostics and the following treatment. Positron emission tomography-computed tomography (PET/CT) is one of the most reliable methods for scanning the whole organism for malignancies. In this work, we synthesized an ^{11}C-labeled oligonucleotide primer and hybridized it to an anti-cancer DNA aptamer. The ^{11}C-aptamer was applied for *in vivo* imaging of Ehrlich ascites carcinoma and its metastases in mice using PET/CT. The imaging experiments with the ^{11}C-aptamer determined very small primary and secondary tumors of 3 mm^2 and less. We also compared ^{11}C imaging with the standard radiotracer, 2-deoxy-2-[fluorine-18]fluoro-D-glucose (^{18}F-FDG), and found better selectivity of the ^{11}C-aptamer to metastatic lesions in the metabolically active organs than ^{18}F-FDG. ^{11}C radionuclide with an ultra-short (20.38 min) half-life is considered safest for PET/CT imaging and does not cause false-positive results in heart imaging. Its combination with aptamers gives us high-specificity and high-contrast imaging of cancer cells and can be applied for PET/CT-guided drug delivery in cancer therapies.

INTRODUCTION

Metastasis formation is the hallmark of cancer and is one of the main reasons cancer patients die. Dissemination of cancer cells through blood circulation and the lymphatic system starts at the early stages of the disease, sometimes even before the primary tumor becomes big enough for its visualization using positron emission tomography (PET), magnetic resonance, or computed tomography (CT).[1] About 3%–5% of all malignancies are cancer of unknown primary origin. Usually, a comprehensive diagnostic examination reveals the primary site; however, it can be expensive, tedious, and invasive. The conventional metastasis detection methods *in vivo* are not sensitive and specific enough to distinguish small volumes of transformed tissues. Therefore, there is a need for an imaging technique capable of precise detection of small primary tumors and metastases that is favorably noninvasive. The use of highly specific radiopharmaceuticals for tumor visualization for PET/CT could facilitate the accuracy of cancer diagnostics and monitoring.[2] PET/CT imaging with radiopharmaceuticals such as 2-deoxy-2-[fluorine-18]fluoro-D-glucose (^{18}F-FDG), ^{11}C-methionine, and ^{11}C-choline is currently one of the most sensitive methods of tumor visualization, determination of the stage, and assessment of the tumor progression.[3–6] Despite the high sensitivity of PET/CT, the radiopharmaceuticals do not demonstrate selectivity, accumulating tumors, and tissues with high metabolic activity.[6] Therefore, the search for novel radiopharmaceuticals capable of targeting tumor cells is of current interest.

Antibodies, peptides, folic acid, and RNA/DNA aptamers could be used as ligands for the radiolabels' targeted delivery to the cells. Antibodies are considered the most popular targeting agents for tumor visualization since almost any imaging probe can be linked to antibodies.[7,8] However, they have several limitations, including low reproducibility.[9] The main reason for antibodies' irreproducibility is variations associated with different manufacturers, different batches from the same manufacturer, low stability at room and body temperatures, and inability to restore an active conformation after protein unfolding.[9,10]

Received 9 February 2021; accepted 19 October 2021;
https://doi.org/10.1016/j.omtn.2021.10.020.

Correspondence: Maxim V. Berezovski, Department of Chemistry and Biomolecular Sciences, University of Ottawa, Ottawa, Canada.
E-mail: maxim.berezovski@uottawa.ca
Correspondence: Anna S. Kichkailo, Krasnoyarsk State Medical University Named After Prof. V.F. Voino-Yasenetsky, Krasnoyarsk, Russia.
E-mail: aszamay@gmail.com

This is an open access article under the CC BY license (http://creativecommons.org/licenses/by/4.0/).

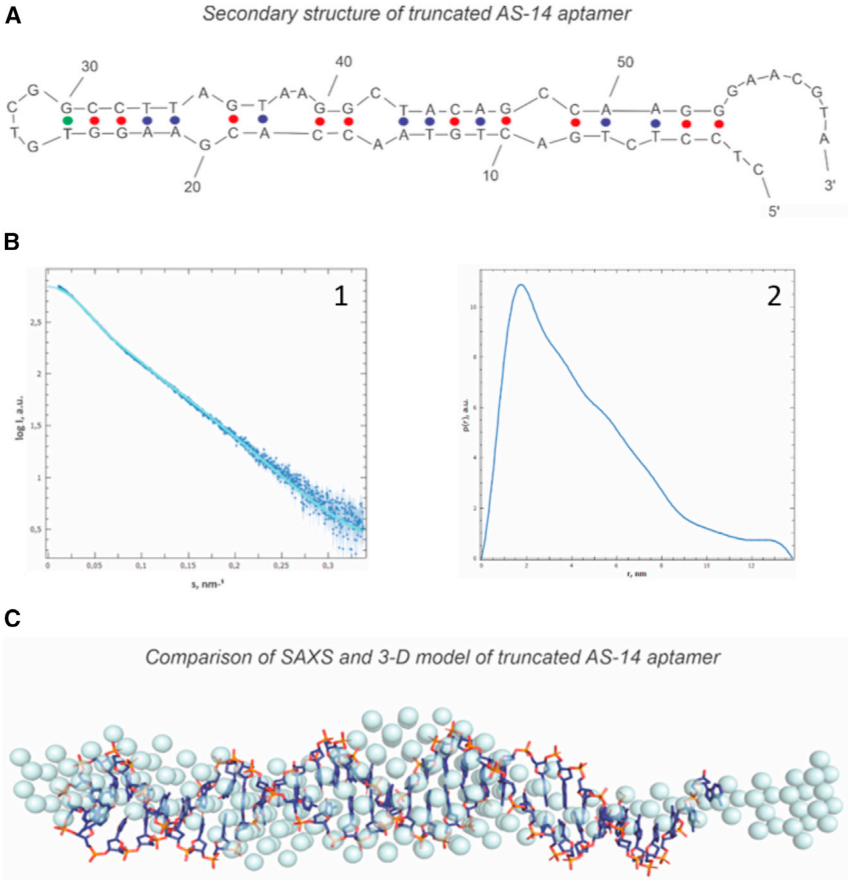

Figure 1. Finding the 3D structure of the truncated AS-14 aptamer

(A) The secondary structure of AS-14t. (B) (1) Experimental SAXS data (dark blue dots) fitted by the theoretical SAXS curve (a light blue line). (2) Pair distance distribution function p (r) of AS-14t. The maximal value of r is the D_{max} (the size of the molecule). (C) Comparing the modeled 3D structures with the experimental SAXS model. Dark blue sticks are from molecular modeling; blue color spheres are from SAXS data.

Aptamers could serve as an alternative to antibodies as targeting, diagnostic, and delivery agents. DNA aptamers are oligodeoxynucleotides of 15–100 nt in length with high affinity and specificity to their targets. The Systematic Evolution of Ligands by Exponential Enrichment (SELEX) technology usually obtains them from a synthetic DNA library with randomized nucleotides.[11,12] The advantages of using aptamers as radiolabeled probes for cancer imaging are specificity to targets and fast clearance in the organism. The aptamer's lifetime in the blood is ideal for PET, allowing large doses of the drug to be administered to the patient, quickly eliminated from the circulatory system without damaging body tissues. High specificity and affinity for the target receptor or cell and the small size (5–25 kDa) of the aptamers ensure good penetration into the tumor and contrast imaging.

Aptamers have been used for targeted delivery of a radioactive label.[13–15] Different radionuclides were attached to aptamers: technetium-99m (^{99m}Tc),[16] indium-111 (^{111}In),[17] copper-64 (^{64}Cu),[18] fluorine-18 (^{18}F).[19]

This study used a radioactive label ^{11}C with ultra-short (20.38 min) half-life that is safe and has simple synthesis. Here we described the preparation and use of a DNA aptamer labeled with a radioactive ^{11}C for *in vivo* imaging of metastases.

RESULTS

Determination of 3D structure of aptamer

The ^{11}C-labeled aptamer probe for *in vivo* tumor and metastasis PET/CT imaging was synthesized based on AS-14 DNA aptamer. This aptamer was previously selected to recognize Ehrlich ascites carcinoma cells.[20] The target protein for the aptamer AS-14 was identified as fibronectin[20] with several post-translational modifications.[21] AS-14 consists of 80 nucleotides, including a 20-nt extension for hybridization with $^{11}CH_3$ primer.

The aptamer's tertiary structure was determined using the small-angle X-ray scattering (SAXS) method (Figure 1). The experimental SAXS curve showed the presence of monomeric AS-14t with a molecular weight of 22 kDa. These data are usually presented in reciprocal space, where the scattering angle is shown in inverse nanometers. The aptamer's structure parameters in solution were derived from the SAXS (Figure 1B1), such as radius of gyration $R_g = 3.6 \pm 0.1$ nm (Figure 1B2), the maximum dimension of the molecule $D_{max} = 13.8$ nm, and average excluded volume $V = 39.5$ nm^3. The structure of the aptamer is an elongate rod-like form. It was confirmed by the distance distribution function p(r), which has a high peak on the small distances (r is about 2 nm) and smaller values on the large distances (Figure 1B). We used the obtained p(r) function represented in real space to reconstruct the molecule's overall shape in solution (Figure 1C), which can be used as the molecular modeling starting point.

The truncated aptamer's primary sequence predicted possible secondary structures using the OligoAnalyzer web server based on free energy minimization techniques. The correct model was chosen based on the experimental data (Figure 1C). The shape of the SAXS model had a length of about 135 Å, and the width at the widest point was about 25 Å, which corresponds to the width of two complementary nucleotides in a double helix. Therefore, the secondary structure model with the most elongated shape and with the largest number of complementary pairs was chosen for further investigation. After computer simulation and optimization, we achieved the theoretical model's complete fitting into the experimental volume (Figure 1C).

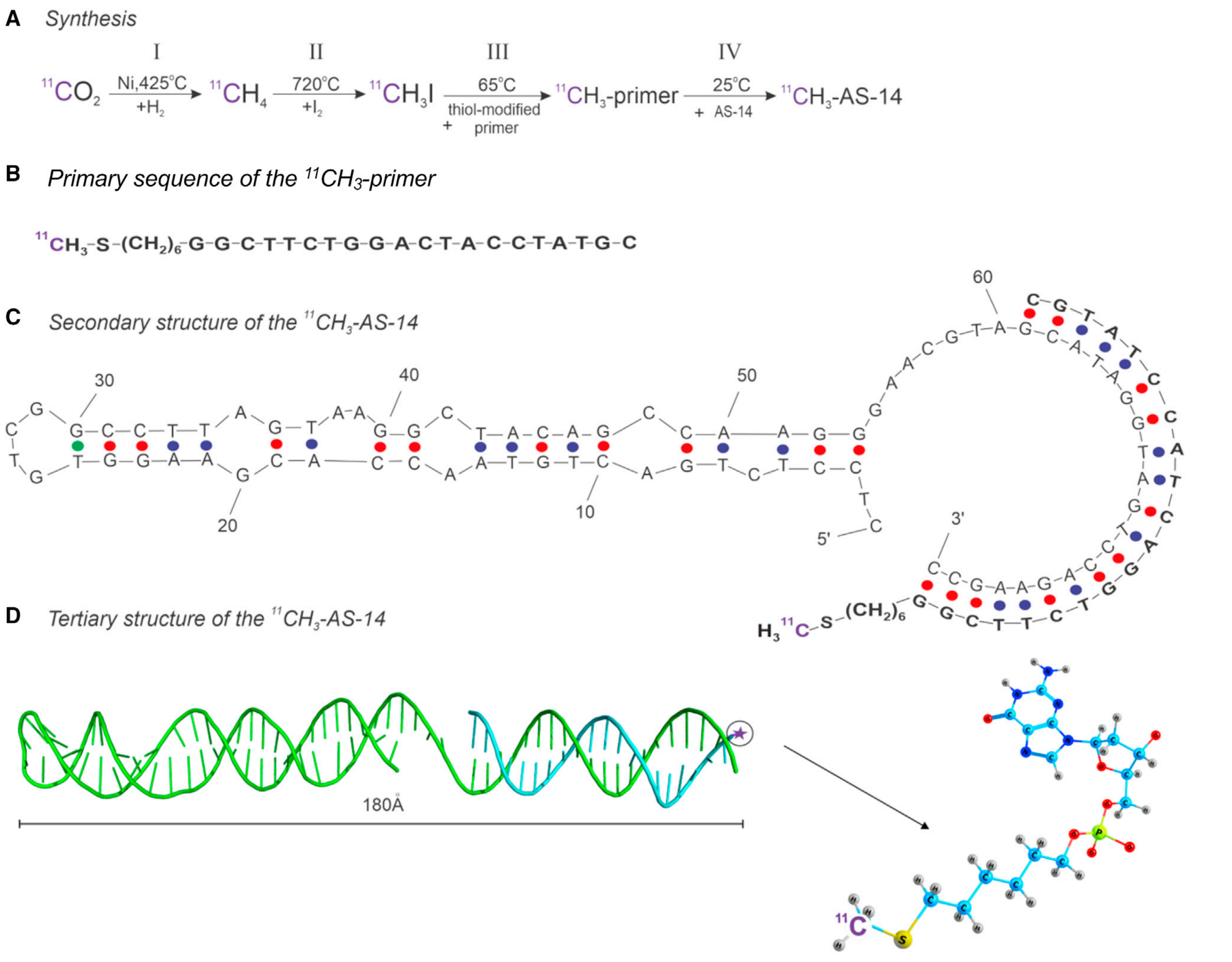

Figure 2. Synthesis of the ^{11}C-labeled aptamer

(A) Synthesis of the $^{11}CH_3$-labeled primer. (B) The primary sequence of the $^{11}CH_3$ primer. (C) Secondary structure of $^{11}CH_3$-AS-14. (D) The tertiary structure of $^{11}CH_3$- AS-14 (green ribbon, AS-14 aptamer; cyan ribbon, $^{11}CH_3$ primer).

Constructed aptamer tertiary structure was used to calculate a theoretical SAXS curve compared with the experimental SAXS data. The discrepancy between both sets of SAXS data χ^2 was 2.812, which shows a good match for the reconstruction.

Synthesis of ^{11}C-labeled radiopharmaceutical

The ^{11}C-labeled radiopharmaceutical was synthesized by the cyclotron (Figure 2A) based on a synthetic 20-nt thiol-modified oligonucleotide (the primer, Figure 2B) complementary to the 3′ end of the aptamer previously selected to Ehrlich carcinoma cells (Figure 2C). The whole procedure is described in the section, "materials and methods." Briefly, radiopharmaceutical synthesis consisted of four steps (Figure 2A): $^{11}CH_4$ synthesis from $^{11}CO_2$, H to I replacement to make $^{11}CH_3I$, the reaction of $^{11}CH_3I$ with the primer's SH group, and the $^{11}CH_3$ primer's hybridization with the aptamer. The resulting radiopharmaceutical has the secondary structure presented in Figure 2C and the tertiary structure predicted by SAXS and molecular modeling in Figure 2D. Molecular modeling demonstrated that the primer and radiolabel did not disturb the functional part of the aptamer and should not decrease the binding abilities.

Evaluation of ^{11}C-primer synthesis efficiency

The efficiency of ^{11}C-primer synthesis was evaluated using horizontal gel electrophoresis. After the synthesis, the radiopharmaceutical was immediately loaded into 2% agarose gel wells and separated for 10 min at 100 V. The electropherogram was analyzed by PET/CT Discovery 600 (General Electrics), which provided a radioactive label visualization (Figure 3A). The ^{11}C-labeled primer moved in an electric field toward the positive electrode because of its negative charge. The free ^{11}C label without DNA remained on the loading well. The

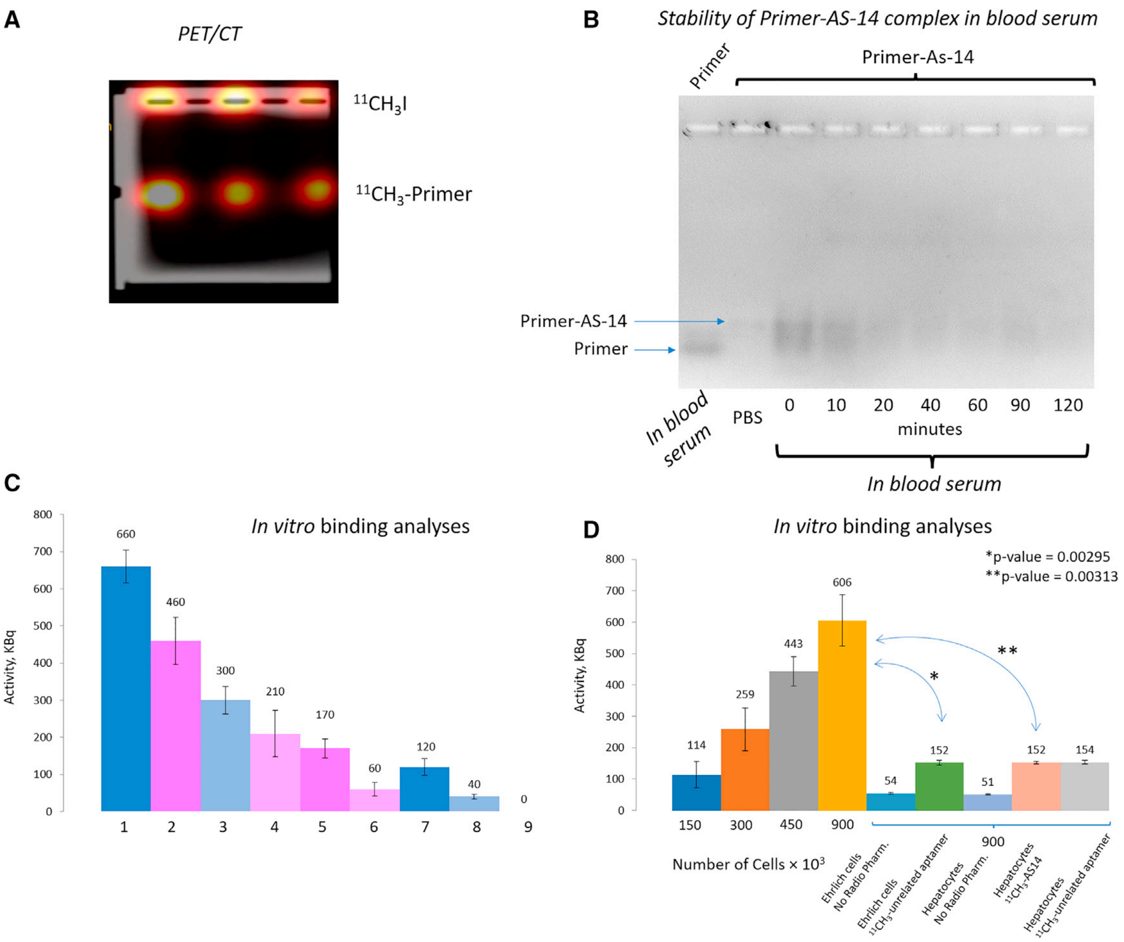

Figure 3. Degradation and binding analyses of ^{11}C-labeled oligonucleotides

(A) Agarose gel electrophoresis of the ^{11}C-labeled primer after synthesis. (B) Agarose gel electrophoresis of the FAM-labeled primer AS-14 in mouse serum at different times. (C) Dependence of the sample's radioactivity on the content of $^{11}CH_3$ primer or $^{11}CH_3$-AS-14, where sample (1) corresponds to 0.6 nmol of $^{11}CH_3$ primer; (2) 0.6 nmol of $^{11}CH_3$ primer hybridized with 0.6 nmol of AS-14; (3) 0.3 nmol of $^{11}CH_3$ primer; (4) 0.3 nmol of $^{11}CH_3$ primer hybridized with 0.3 nmol of AS-14 ($^{11}CH_3$-AS-14); (5) 0.6 nmol of $^{11}CH_3$ primer hybridized with 0.6 nmol of AS-14 ($^{11}CH_3$-AS-14) incubated with Ehrlich cells; (6) 0.3 nmol of $^{11}CH_3$ primer hybridized with 0.3 nmol of AS-14 ($^{11}CH_3$-AS-14) incubated with Ehrlich cells; (7) 0.6 nmol of $^{11}CH_3$ primer incubated with Ehrlich cells; (8) 0.3 nmol of $^{11}CH_3$ primer incubated with Ehrlich cells; (9) Ehrlich cells only. (D) Correlation of the radioactivity of the samples versus the number of ascites cells or hepatocytes bound with $^{11}CH_3$-AS-14 or the $^{11}CH_3$-unrelated aptamer. Errors bars are one SD of three measurements.

average activity value obtained for the DNA-free $^{11}CH_3I$ at the starting wells was 542 ± 72 kBq/mL. The activity of the ^{11}C-labeled DNA was 380 ± 87 kBq/mL (Figure 3A). These data indicated that around 42% of ^{11}C was attached to DNA during the radiosynthesis. In starting wells, DNA was not detected (using NanoDrop 2000, Thermo Fisher Scientific, United States), and the average amount of DNA in the low bands was 0.022 ± 0.005 nmol. The volume of the radiopharmaceutical was 2 mL; therefore, the final concentration of the ^{11}C primer was 2 μM. The average activity of the resulted radiopharmaceutical was 190 × 10^{12} Bq/mol.

Evaluation of $^{11}CH_3$-AS-14 binding with ascites cells in vitro

Before *in vivo* analyses, we estimated the stability of the primer-AS-14 complex in mouse blood serum at 37°C. Electropherogram of the complex incubated in undiluted mouse serum showed the degradation of the complex over time. Comparison of the intensities of the primer-AS-14 complex demonstrated that, after 10 min of incubation, almost 50% of primer-AS-14 was degraded; after 40 min, 39% of the complex was left. The 120-min incubation in blood serum resulted in 80% primer-AS-14 degradation (Figure 3B). Thus, the PET/CT imaging with the aptamer was performed 30–40 min after injection. The *in vitro* activity of $^{11}CH_3$-AS-14 was assessed in two series of experiments (Figures 3C and 3D). In the first series, the $^{11}CH_3$-AS-14 radioactivity was assessed depending on its concentration. Cells were incubated for 10 min at 37°C on a shaker (longer incubation decreased radioactivity due to the radionuclide decay). Phosphate buffer, aptamers, and ascites cells were used as dilution factors. Studies showed that the sample radioactivity (determined using a

Table 1. Comparative analysis of specific detection with different radioactive probes such as $^{11}CH_3$-AS-14, ^{18}F-FDG, $^{11}CH_3$ primer, and $^{11}CH_3$-unrelated aptamer on mice with metastases in different organs obtained using PET/CT and confirmed by autopsy and histological examination

Organ	The number of metastases detected by different methods			
	PET/CT with $^{11}CH_3$-AS-14/autopsy and histological examination (N = 6 mice)	PET/CT with $^{11}CH_3$ primer/autopsy and histological examination (N = 5 mice)	PET/CT with $^{11}CH_3$-unrelated aptamer/autopsy and histological examination (N = 4 mice)	PET/CT with ^{18}F-FDG/autopsy and histological examination (N = 2 mice)
Liver	6/6	low irradiation 5/5	low irradiation 5/5	0/2
Tumor nodes	3/3	0/2	0/0	0/1
Thymus and Heart	3/3	0/2	0/1	non-specific accumulation/2
Bladder	0/1	0/1	0/0	0/0
Intestines	4/4	0/2	0/2	0/2
Kidneys	3/3	low irradiation 5/3	0/3	low irradiation 2/2
Lungs	2/3	0/2	0/3	low irradiation 1/1
Spleen	1/1	0/1	0/0	0/0
Thyroid gland	2/2	0/2	0/2	2/2
Stomach	1/1	0/0	0/1	0/1
Muscles	2/2	0/0	0/0	1/1
Pancreas	1/1	0/0	0/0	0/1
Bone marrow	1/1	0/0	0/0	0/1

dosage calibrator [Comecer Petagora, Italy]) varied linearly depending on the content of the ^{11}C radionuclide in it (Figure 3C).

In the second series of experiments, ascites cells' radioactivity after their binding to $^{11}CH_3$-AS-14 was evaluated. The ascites cells preincubated with masking RNA were incubated with $^{11}CH_3$-AS-14 for 10 min, then washed twice to remove the unbound aptamers. After that, samples with different cell contents were prepared, and the radioactivity in the samples was determined using a dosage calibrator (Figure 3D). The radioactivity depended on the number of cells in the sample; the highest value was reached for the highest cell number (900,000 cells). The same number of cells were used in control analyses with an unrelated aptamer and hepatocytes. $^{11}CH_3$-AS-14 and the unrelated aptamer did not bind to hepatocytes (Figure 3D). The unrelated aptamer did not bind to Ehrlich ascites cells (Figure 3D).

Evaluation of $^{11}CH_3$-AS-14 binding with ascites cells *in vivo*

The ability of $^{11}CH_3$-AS-14 to find and detect tumor foci and metastases in the body was assessed on Imprinting Control Region (ICR) mice with Ehrlich tumors. Metastatic tumor models were used in the study. Metastases were formed by intravenous injection of 0.3 million Ehrlich cells into the tail vein of mice. At the first series of the experiment (9 days after intravenous injection of tumor cells to mouse tail veins), the time of PET/CT scanning was adjusted. The scanning was performed 10, 20, 30, and 40 min after intravenous injection of $^{11}CH_3$-AS-14 (Figure S1). Ten and 20 min were not enough for $^{11}CH_3$-AS-14 to give a contrast image; the aptamer did not concentrate in the tumor foci. The highest contrast was achieved 40 min after injection of the radiopharmaceutical (Figures S1A4 and B4). Therefore, all the following measurements were performed after 30 and 40 min of injection. Nine days after tumor intravenous transplantation, mice had metastases in the liver, kidneys, and spleen. We note that irradiation on mouse tails was an artifact from the undistributed radiopharmaceuticals (Figures 5, 6, and S1).

Sixteen days after tumor transplantation, the mice with random metastases and control healthy mice were imaged using PET/CT scanning 40 min after intravenous injection of $^{11}CH_3$-AS-14 conjugate or $^{11}CH_3$ primer, $^{11}CH_3$-unrelated aptamer, and ^{18}FDG as control radiopharmaceuticals. The radiolabeled aptamer allowed the identification of metastases in different mouse organs such as lungs, thymus, intestines, heart, and spleen (Table 1; Figures 4, 5, S2, and S3). The highest irradiation was observed in tumor-affected liver, spleen, kidneys, and sizable nodular lesions in the abdominal cavity (Figures 4 and S3). For each metastasis, the location was proved using autopsy and histological analyses (B and C in Figures 4, 5, and S3). Metastatic lesions were easily distinguished both macroscopically (Figure 4B) and microscopically (Figure 4C). The metastatic lesion in each organ was characterized by proliferating tumor cells with pronounced phenomena of cytological atypia, expressed in the form of nuclear polymorphism, nuclear hyperchromasia, and violation of the nuclear-cytoplasmic ratio (Figure 4C). Tumor cells contained a significant number of pathological mitoses, large basophilic nucleoli (Figure 4C). The large foci of tumor growth were mainly found in the livers, and tumor nodes in abdominal cavity necrosis were manifested in the form of geographic coagulation with cell detritus (Figures 4C, 5D, and S3C). The Discovery PET/CT 600 scanner (General Electric, United States) has an 8-mm^2 resolution limit for the standard radiopharmaceuticals such as ^{18}FDG and methionine. Here we noticed that, in the case of ^{11}C-aptamer, the resolution was much higher (<2 mm^2), so we were able to distinguish even smaller metastases

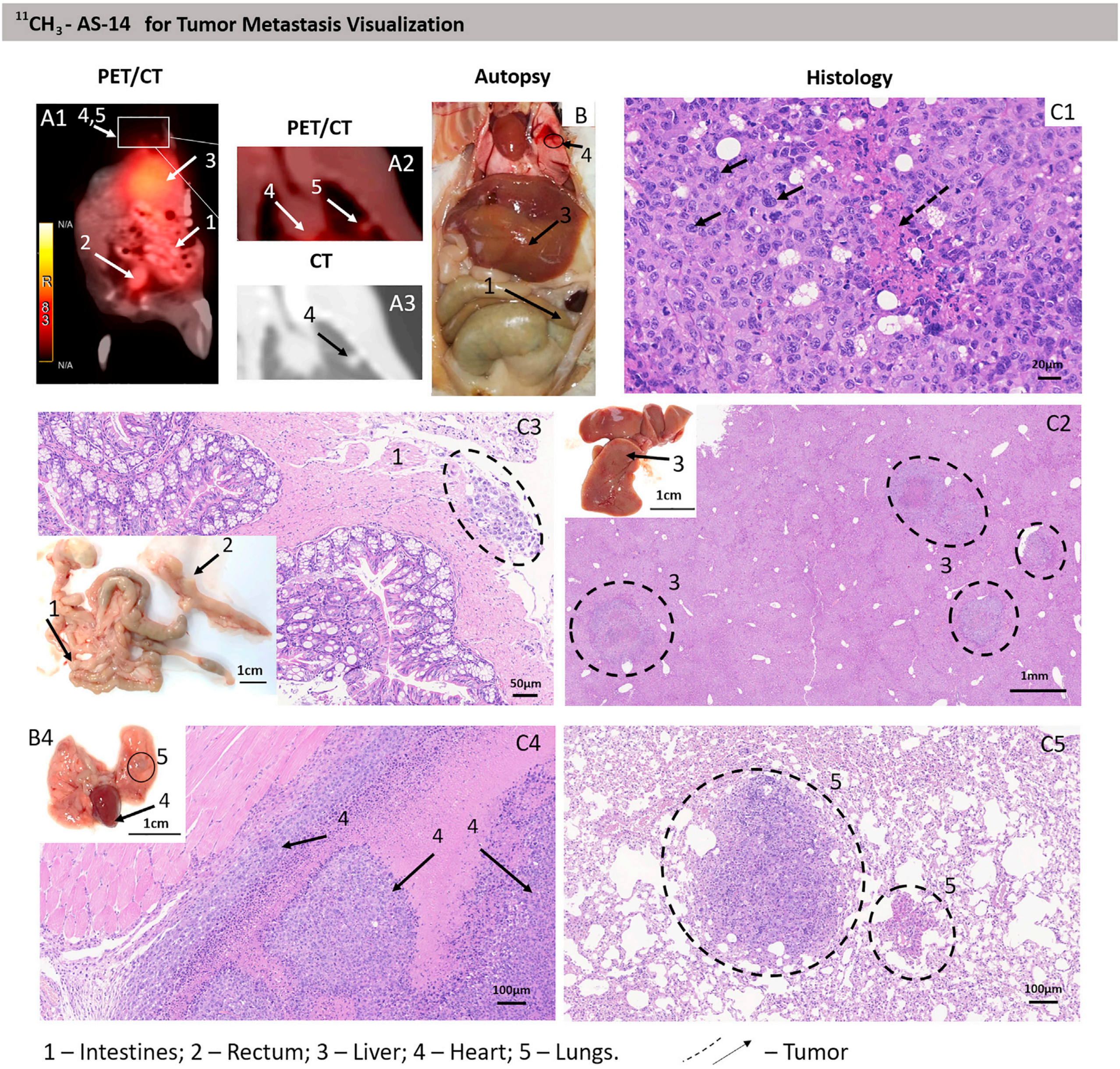

Figure 4. Tumor and metastases localization analysis using the $^{11}CH_3$-AS-14 probe and PET/CT

(A–C) The representative PET/CT images of mice with metastases (A) were confirmed by autopsy (B) and histological analyses of metastatic tumor lesions (C) in different organs: intestines (2); liver (3); heart (4); and lung spleen (5). Cytological characteristics of the tumor tissue (C). Hematoxylin-eosin staining. Magnification C1, ×400; C2, ×200; C3, ×30; C4 and 5, ×100. Arrows and dashed circles indicate the tumor sites in different organs at PET/CT, autopsy, and corresponding tissue sections.

(Figures 5A1 and A2) compared with ^{18}FDG (Figures 5B1 and B2). The aptamer-based radiopharmaceutical distinguished small metastasis spread all over the body in thyroid glands, stomach, liver, kidney, intestines, muscles, lungs, heart, pancreas, and even bone marrow of the ribs (Figures 5A1, A2, and D1–D9). ^{18}F-FDG was accumulated in the chest, throat, and along the spine (Figure 5B1) and did not allow identifying localization of the small metastasis. Mice with metastasis and injected with $^{11}CH_3$ primer or an unrelated aptamer as controls demonstrated similar physiological distribution of the oligonucleotide-based radiopharmaceuticals with slight irradiation in the liver (Figures 6A and 6B). The same distribution was observed in healthy mice injected with $^{11}CH_3$-AS-14 (Figure 6C). ^{18}F-FDG was accumulated in the heart and active muscles but not in the liver (Figure 6E). It is important to note that oligonucleotide-based radiopharmaceuticals and ^{18}F-FDG were excreted with urine, and the full bladder had high irradiation (Figures 6A1, E [arrow 5], S1, and S2).

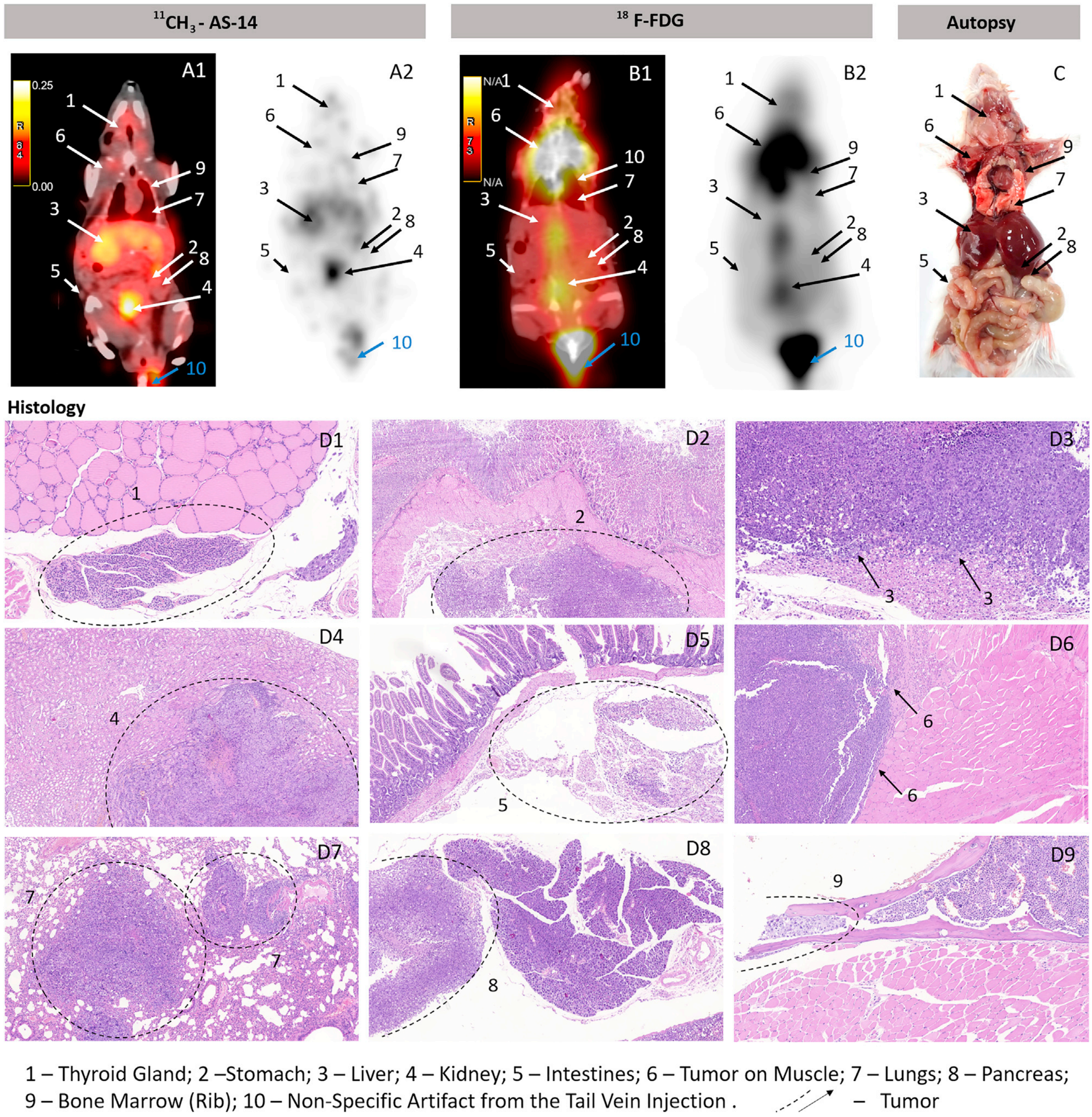

Figure 5. Comparative PET/CT imaging using $^{11}CH_3$-AS-14 with ^{18}F-FDG

(A–D) Images with $^{11}CH_3$-AS-14 (A1 and 2) and ^{18}F-FDG (B1 and 2) on the same mice scanned on the next day. Accuracy of PET/CT results was confirmed by autopsy (C) and histological analyses of metastatic tumor lesions (D) in different organs: thyroid gland (1); stomach (2); liver (3); kidney (4); intestines (5); muscle (6); lung (7); pancreas (8); rib bone marrow (9). Arrows and dashed circles indicate the tumor sites in different organs at PET/CT, autopsy, and corresponding tissue sections.

It is important to note that, even though we used the same scanning protocol for mice injected with ^{18}F-FDG and the aptamer-based radiopharmaceutical, the images of $^{11}CH_3$-AS-14 mice had a much higher resolution (Figures 5A and 5B; Video S1). Moreover, it was observed that ^{18}F-FDG accumulated in metabolically active organs, such as the heart; therefore, it cannot be useful for searching metastases in the heart. We were able to distinguish the smallest metastatic lesions all over the body, even in the heart, lungs, intestines, thymus, and ribs (Figure 6A). All tumor sites were proved in autopsy. In all cases, the metastatic growth of the tumor was confirmed by

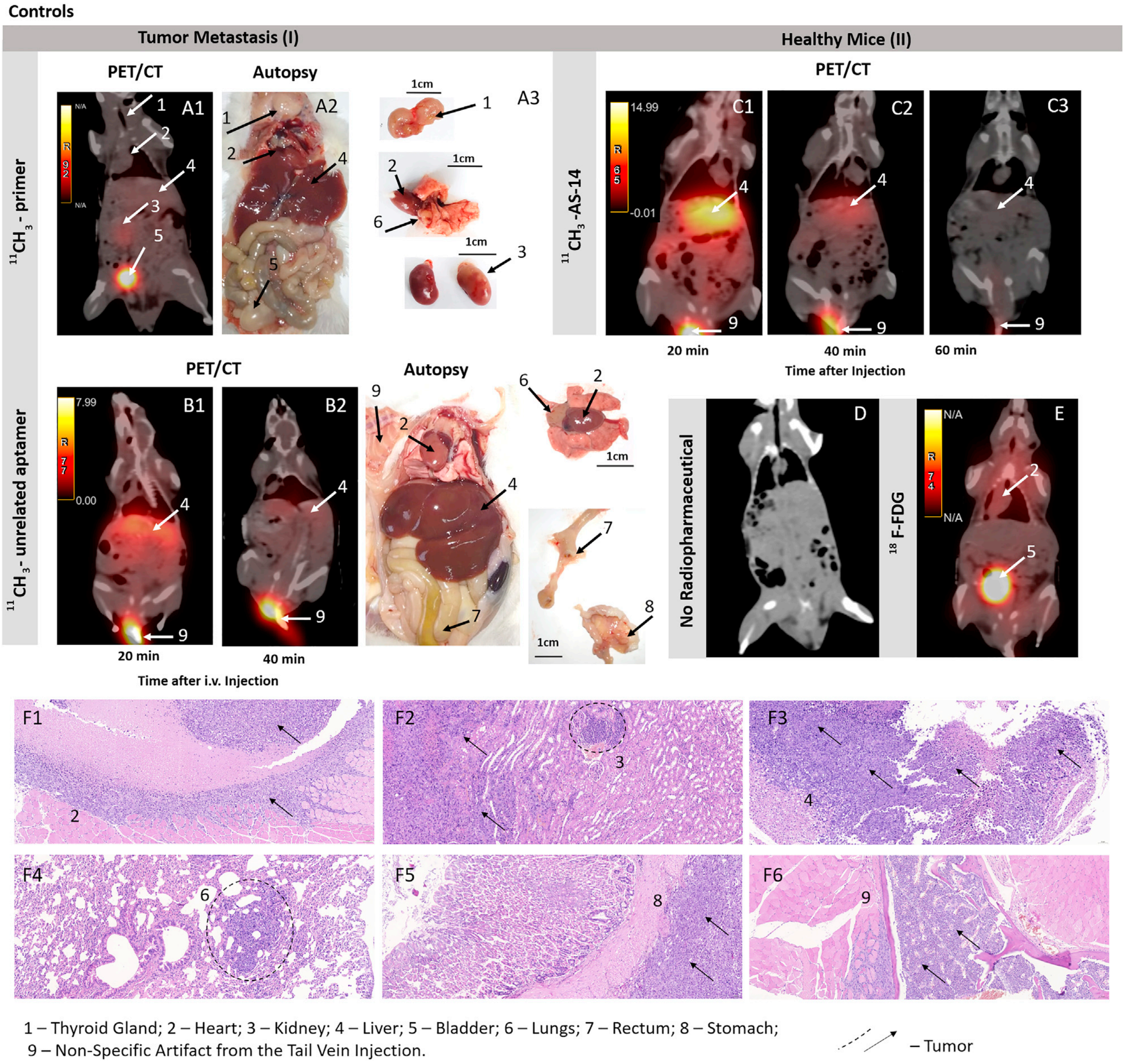

Figure 6. Specific recognition of the tumor sites by $^{11}CH_3$-AS-14 proved by control experiments

(A–F) The representative PET/CT analysis of mice with tumor metastasis (I) using the $^{11}CH_3$ primer (A) and $^{11}CH_3$-unrelated aptamer (B) as probes. Metastasis formation was confirmed by autopsy (A2, B3). PET/CT images of physiological distribution and excretion of $^{11}CH_3$-AS-14 (C) and ^{18}F-FDG (E) in healthy mice (II). PET/CT image of a healthy mouse without the injected radiopharmaceutical (D). Histological examination with the staining of tissue sections with hematoxylin-eosin (F). Arrows indicate the tumor sites in different organs at PET/CT and autopsy.

histological examination with the staining of tissue sections with hematoxylin-eosin (Figure 6F). Sometimes we could not distinguish metastatic lesions in the heart and spleen or rib bone marrow macroscopically at autopsy, but histological analyses proved the tumor localization in these organs.

Table 1 compares data on metastasis localization obtained using PET/CT, autopsy, and histological analysis. According to PET/CT imaging with $^{11}CH_3$-AS-14, all five mice were metastatic. These observations were proved by autopsy and histological examinations. $^{11}CH_3$ primer and $^{11}CH_3$-unrelated aptamer demonstrated low irradiation in mouse livers. Three mice had tumor nodes in the abdominal cavity, which were identified using PET/CT, macroscopic and microscopic observations. Tumors in various organs were detected by PET/CT and proved by autopsy. It was impossible to distinguish tumors in the bladder because the radiopharmaceutical was excreted with the urine.

Acute toxicity of $^{11}CH_3$-AS-14

PET/CT scanning of the healthy mice injected with $^{11}CH_3$-AS-14 showed its non-specific accumulation in the liver and other abdominal organs (Figure 6C1 and 6C2) and it was entirely excreted within 60 min (Figure 6C3). Our acute toxicity study demonstrated that the aptamer-based radiopharmaceutical was not toxic. Injected intramuscularly, $^{11}CH_3$-AS-14 was nonspecifically accumulated in different organs. Its radioactivity measured using a dosage calibrator was detected in mouse organs only 20 min after administration. The dosage of radioactivity accumulated in the organ was normalized per 1 g of the tissue (Figure S4A). Residual radioactivity in the muscle where the injection was made was not detected. This data support the PET/CT data on the rapid excretion of the radiopharmaceutical within 20 min.

The toxicity of $^{11}CH_3$-AS-14 was assessed by the changes in blood biochemical parameters: levels of total protein and bilirubin; activities of alanine aminotransferase, alkaline phosphatase, and alpha-amylase. These indicators were chosen because they reflect functions of the pancreas, kidneys, and liver. Total plasma proteins and albumin describe the protein exchange in the organism and liver. The loss of albumin in the urine shows a problem with blood filtration in the kidneys. The content of total protein in the mouse serum after 20 min, 40 min, and 24 h of administering the radiopharmaceutical was not different from control mice (Figure S4B).

Bilirubin is a bile pigment formed due to the breakdown of proteins containing heme (hemoglobin, myoglobin, cytochrome). Bilirubin increase indicates both excessive destruction of erythrocytes (hemolytic jaundice, etc.) and impaired excretion of bilirubin from the body. In this experiment, a slight increase in bilirubin 40 min after injection was observed, but this increase was statistically insignificant (Figure S4C).

Amylase is a digestive enzyme predominantly secreted by the pancreas and salivary glands and is found in minimal amounts in other tissues. A change in amylase activity can be observed in the case of poisoning, dysfunction of the pancreas and salivary glands, and renal failure. $^{11}CH_3$-AS-14 administration did not change amylase activity significantly (Figure S4D).

Alkaline phosphatase belongs to the enzymes found in almost all body tissues, with predominant localization in the liver, bones, and placenta. The activity of total alkaline phosphatase increases when liver, bones, and kidneys tissues are damaged. In our experiments, alkaline phosphatase activity was normal. It indicated that all organs mentioned above stayed safe after $^{11}CH_3$-AS-14 administration (Figure S4E). Alanine aminotransferase belongs to a subgroup of aminotransferases (transaminases). It is widely used in medical practice for laboratory diagnosis of liver damage. The alanine aminotransferase activity did not change significantly after $^{11}CH_3$-AS-14 administration compared with a control (Figure S4F).

Histological analyses of the corresponding mice did not reveal any differences between the groups. In liver tissue, the histological structure was preserved (Figure S5A). Portal tracts were without signs of sclerosis, with focal minimal lymphoplasmacytic infiltration, limited by the stroma of the portal tract, which does not extend to the parenchyma. There were no signs of inflammation or sclerosis in the bile ducts and cholestasis. The central veins contained red blood cells or were empty, not dilated. In the parenchyma of the hepatic lobules in the cytoplasm of hepatocytes, small and medium-sized vesicles were diffusely determined. Vesicles were primarily located along the cell's periphery, at the basement membrane, and sometimes in the center, closer to the nucleus. The nuclei of hepatocytes of different sizes were located in the center of the cells, with several small nucleoli. Hepatocyte balloon degeneration was not detected. Single scattered hepatocytes were in apoptosis or necrosis. The number of Kupffer cells was not increased. The same histological picture was repeated in tissue sections of all mice from all four groups (Figure S5A).

Administration of $^{11}CH_3$-AS-14 did not influence kidney morphology (Figure S5B). In kidney tissue, the glomeruli were of normal size and cellularity. Sclerosed glomeruli were not defined. There were no zones of tubular atrophy. The epithelium of the tubules was preserved; the nuclei were of the same size, with hardly prominent nucleoli; the cytoplasm was eosinophilic, non-granular. In scattered epithelial cells, single intracytoplasmic vacuoles were determined. There were no signs of inflammation or sclerosis. The lumen of the vessels had erythrocytes (Figure S5B).

DISCUSSION

Synthesis of novel radiopharmaceuticals for cancer metastasis is of great importance because the existing radionuclides for cancer imaging do not demonstrate enough selectivity, accumulating in tumors and tissues with high metabolic activity. An ideal radiolabeled molecular probe for PET/CT for cancer diagnosis should be sensitive enough to detect even small metastases, specific, and capable of clearly delineating the tumor volume. Simultaneously, it should have an ultra-short half-life to clear from the body rapidly, and facilitate early image acquisition, because it is safer for the patient. Radiolabels could be delivered to the tumor sites by carriers such as nanoparticles,[22] antibodies,[23] tyrosine kinase inhibitors,[24] albumins,[25,26] and peptides.[27,28] DNA and RNA aptamers, along with antibodies and peptides, could be used as ligands for the targeted delivery of the labels to the cells. Some successful attempts have shown that radionuclides could be used to label aptamers: ^{99m}Tc,[16] ^{111}In,[17] ^{64}Cu,[18] ^{18}F.[19] Nonetheless, to date, there is no simple universal technology for the synthesis of radiolabeled aptamers. It is challenging to obtain a stable target product with high radiochemical purity.

Currently, ^{11}C-radiopharmaceuticals have gained increased importance in clinical PET for cancer diagnosis for several reasons. Labeling with ^{11}C does not influence the biomolecule's chemical structure and properties. ^{11}C is considered one of the safest radionuclides for PET chemistry. Carbon, oxygen, hydrogen, sulfur, phosphorus, and nitrogen are the basic elements of organic molecules in living organisms. Radiolabeling with ^{11}C makes the radiopharmaceutical indistinguishable from its stable natural counterpart. Thus, it can be used as a true tracer

to investigate biological processes without influencing their rate or outcome to any measurable extent. Another important aspect is the ease of its synthesis and small influence on the attached molecule. Here we described in detail the radiopharmaceutical synthesis of ^{11}C-labeled aptamer, demonstrating that the labeling did not influence the aptamer's conformation and binding properties. It is important that the ^{11}C-radiolabeled aptamer detected even the smallest tumor sites less than 2 mm^2, demonstrating better resolution than standard ^{18}F-FDG. $^{11}CH_3$-AS-14 was excreted rapidly and not toxic for off-target organs. It has been demonstrated that aptamers are highly selective and accumulated in most tumors spread in the organism; therefore, it opens new cancer diagnostic modalities and therapy. Radiolabeled aptamers could be used for PET/CT-guided drug delivery.

MATERIALS AND METHODS

The ^{11}C-radiolabeled aptamer consists of AS-14 aptamer 5′-CTC CTC TGA CTG TAA CCA CGA AGG TGT CGG CCT TAG TAA GGC TAC AGC CAA GGG AAC GTA *GCA TAG GTA GTC CAG AAG CC*-3′ with an extended part (in italic) and a complimented primer ($^{11}CH_3$ primer) 5′-$^{11}CH_3$-S-(CH2)$_6$-GGC TTC TGG ACT ACC TAT GC-3′ to the extended sequence at the 3′ end of AS-14.

Synthesis of oligonucleotides

Oligonucleotide synthesis was performed on an ASM-800 DNA/RNA synthesizer (Biosset, Russia) on a 0.1 μM scale. Oligonucleotides were synthesized using standard phosphoramidite (dA^{Bz}, dC^{Ac}, dG^{ibu}, T ChemGenes, United States; 5′-thiol-modifier C6 Glen Research, United States). The HS-$(CH_2)_6$ group was attached at the oligonucleotide 5′ end. The oligonucleotide was deprotected and removed from the solid support using concentrated NH_3 (aq).

Purification was accomplished using RP-Cartridge (ChemGenes) according to standard manufacturer's protocols. The final deblocking of the oligonucleotide involved the cleavage of the trityl-sulfur bond. This was accomplished by oxidation with silver nitrate, with the excess silver nitrate being precipitated with dithiothreitol (DTT). Excess DTT was removed by extraction with ethyl acetate.

Determination of 3D structure of the aptamer using SAXS

To determine the shape of the aptamer AS-14 without the extended part (AS-14t) in a phosphate buffered saline (PBS) (with Ca and Mg) solution, the SAXS method was applied. The measurements were carried out on the beamline P12 BioSAXS, EMBL (European Molecular Biology Laboratory) at the Deutsches Elektronen-Synchrotron (DESY) center (Hamburg, Germany). The beamline X-ray wavelength was 0.12 nm; sample to detector distance, 3.0 m; sample temperature, 20°C; and single-exposure time, 1 s. The SAXS data acquisition was performed in the size-exclusion chromatography (SEC)-SAXS mode[29] using high-pressure liquid chromatography (HPLC) column Superdex 75 Increase 10/300 GL at a pressure of 11.8 bar and flow rate 0.4 mL/min. During the SEC regime, 3,600 counts of SAXS expositions were recorded by the detector Pilatus6M. The SAXS data from the aptamer molecule in solution were processed according to the standard pipelines.[30,31] To manipulate and process the SAXS data, the program PRIMUS[32] was implemented into the program suite ATSAS.[33] Indirect Fourier transform of the SAXS curve yield the intramolecular distance distribution function p(r) calculated in the GNOM program.[34] The bead model of the aptamer's overall electron density consisting of uniform spheres was reconstructed utilizing the DAMMIN program.[35]

The secondary structure was predicted using the OligoAnalyzer web server.[36] Folding simulations were performed according to the same conditions as the SAXS experiment with the presence of Na^+ (146 mM), Ca^{2+} (0.5 mM), and Mg^{2+} (0.5 mM) ions at 20°C and 37°C. OligoAnalyzer yielded many possible secondary structures at these conditions, from which one model was chosen for further investigations. The tertiary structure of AS-14t was modeled with the Avogadro[37] according to the predicted secondary structures' schemes. The obtained atomic structure was optimized using the fragment molecular orbital method (FMO)[38] in the density functional-based tight binding (DFTB).[39] The solvent effects were described within the polarizable continuum model (PCM).[40] Quantum chemical calculations were carried out using the GAMESS program.[41]

To validate the reconstructed tertiary structure, a theoretical SAXS curve from the molecular model was fitted to the experimental data by the CRYSOL program[42] through the SASpy plugin[43] in the PyMOL program.[44] This program allows one to make the "simulated annealing" of the molecular model, taking into account all atoms' positions in the molecule and calculating the resulting SAXS pattern from the whole molecule, including the hydration envelope surrounding the aptamer in solution.

Synthesis of the ^{11}C-labeled primer

The synthesis of ^{11}C was performed based on the 20-nt primer complimentary to the 3′ end of the aptamer AS-14 on the cyclotron, Cyclone 18/9 ST (IBA, Belgium). The synthesis of the radiolabeled oligonucleotide is schematically represented in Figure 2A. Pure nitrogen gas of natural isotopic composition with oxygen gas (0.5%–1%) was used as a target substance. The resulting ^{11}C was stabilized with oxygen to form $^{11}CO_2$. Purified $^{11}CO_2$ was reduced to $^{11}CH_4$ by hydrogen H_2 in the presence of Ni catalyst in a furnace at 425°C. Reduced $^{11}CH_4$ was fed into the circulation system, which contained iodine vapor. The resulting compound was methyl iodide, $^{11}CH_3I$. The introduction of ^{11}C into the complimented primer to the extended sequence at the 3′ end of AS-14 modified with 5′-thiol modifier C6 S-S phosphoramidite was carried out in a dipolar aprotic solvent, which supported the second-order nucleophilic reactions of DMSO. For this, the reaction mixture was prepared: 40 μL of the primer (100 μM) was added to 500 μL of DMSO, 10 μL of 0.1 M hydrochloric acid solution, 1,500 μL of PBS, pH 7.4 (136.8 mM NaCl, 10.1 mM Na_2HPO_4, 2.7 mM KCl, 1.8 mM K_2HPO_4, 0.499 mM $MgCl_2$), and transferred to the reaction vessel of the automated Synthra MeIPlus synthesis module (Synthra, Germany). $^{11}CH_3I$ was transferred to a reaction vessel with a reaction mixture. At this stage, the primer with a disulfide group reacted with $^{11}CH_3I$ at 65°C for 15 min in a closed reaction vessel. The final volume of 2 mL of

radiopharmaceutical contained 4 nmol of the $^{11}CH_3$-modified primer. The $^{11}CH_3$ primer in Dulbecco's Phosphate Buffered Saline (DPBS) was stable for 60 min.

To assess the efficiency of ^{11}C radionuclide synthesis, we used 2% agarose horizontal gel electrophoresis. For that, 12, 9, and 6 μL of the ^{11}C primer were added to the wells of the agarose gel, after which the gel was placed in a gel electrophoresis system (Advance Mupid-One, Belgium) for 10 min at 100 V. Each sample was prepared in three replicates. Then the agarose gel was scanned at combined PET/CT tomograph Discovery 600 (General Electrics), determining the bands of radiopharmaceutical on the gel. Activity values of the bands were determined using the PET VV software package of the AW Volume Share workstation. DNA-free $^{11}CH_3I$ remained on loading wells, and the ^{11}C primer moved to a positive electrode. The effectivity was calculated as a portion of the activity of the radiopharmaceutical bound with the oligonucleotide. The irradiation bands were cut out from the gel, placed in 1.5-mL tubes, and the next day DNA was extracted using MinElute PCR Purification Kit (QIAGEN, Germany). The DNA concentration in the resulting radiopharmaceutical was measured on NanoDrop 2000 (Thermo Scientific, United States).

Hybridization of ^{11}C primer with AS-14 aptamer

AS-14 aptamer was denatured by heating to 95°C for 10 min, mixed with the equimolar concentration of the $^{11}CH_3$ primer for hybridization, then cooled on ice to restore 3D conformation.

Cell culture

Mouse Ehrlich ascites cells were cultured in 35 × 10-mm cell culture dishes (CELLSTAR, Germany) in Dulbecco's modified Eagle's medium (DMEM; Sigma-Aldrich), supplemented with 100 U/mLpenicillin, 100 U/mL streptomycin, and 5% (v/v) fetal bovine serum (FBS) in a humidified atmosphere containing 5% CO_2 at 37°C. All cell experiments were performed in PBS, pH 7.4, containing 136.8 mM NaCl, 10.1 mM Na_2HPO_4, 2.7 mM KCl, 1.8 mM $K2HPO_4$, and 0.49 mM $MgCl_2$.

Evaluation of primer-AS-14 stability in mouse serum

Stability of primer-AS-14 in undiluted fresh mouse serum was evaluated using 2% agarose horizontal gel electrophoresis after 0, 10, 20, 40, 60, 90, and 120 min of incubation at 37°C. For that, a 5-μL complex of the 6-Carboxyfluorescein (FAM)-labeled primer with the FAM-labeled aptamer AS-14 in blood serum was added to the wells of the agarose gel. The FAM-labeled primer in PBS was taken as a reference molecule. The gel was placed in a gel electrophoresis system (Advance Mupid-One, Belgium) for 20 min at 100 V; the fluorescence was evaluated using a fluorescent gel documentation system G-Box (Syngene, Cambridge, UK). Degradation was estimated by decreasing the band intensity along incubation time in mouse blood serum, evaluated using the software GeneTools (Syngene, Cambridge, UK). The portion of the band corresponding to the primer-AS-14 complex at 0-min condition was taken as 100%. The portion of degradation was calculated after 10, 20, 40, 60, 90, and 120 min of incubation in mouse blood serum.

Binding evaluation of $^{11}CH_3$-AS-14 with tumor cells *in vitro*

Mouse Ehrlich ascites cells in PBS were incubated on a shaker at room temperature for 30 min with yeast RNA (final concentration 0.01 ng/mL) to mask non-specific binding sites. $^{11}CH_3$-AS-14 or $^{11}CH_3$ primer at final concentrations of 150 nM and 300 nM were incubated with 300,000 cells in 500 μL of PBS for 30 min on a shaker at 37°C. After the incubation, unbound radiopharmaceuticals were removed by centrifugation, after which the cells were washed twice with PBS. Then, 500 μL of PBS was added to the cell pellet. The cells were mixed on a shaker. Each sample was prepared in three replicates.

Cell titration experiments were performed with $^{11}CH_3$-AS-14 (final concentration 150 nM). The samples contained 150, 300, 450, and 900 $\times 10^3$ cells. The radioactivity of the obtained samples was measured using a dosage calibrator (Comecer Petagora, Italy).

Binding evaluation of $^{11}CH_3$-AS-14 with tumor cells *in vivo*

The protocol was approved by the Local Committee on the Ethics of Animal Experiments of the Krasnoyarsk State Medical University (number #77 from June 26, 2017). This study was carried out according to the recommendations in the Guide for the Care and Use of Laboratory Animals of the National Institutes of Health. All procedures were performed under anesthesia, and all efforts were made to minimize the suffering of animals. White 6-week-old female 25-g ICR mice were provided by Vector (Novosibirsk, Russia).

To form metastasis, suspension of 0.3 million Ehrlich ascites carcinoma cells in 50 μL was injected intravenously into a mouse tail vein. Tumor cells circulating in the blood formed metastases randomly in different organs: lungs, lymph nodes, kidneys, liver, thymus, heart, thymus, abdominal cavity, intestines, and testicles. On days 9 (n = 4) and 16 (n = 6), when metastases had been formed, the mouse tumors were visualized using PET/CT. The next day after PET/CT, the mice were sacrificed with the subsequent autopsy and tissue histological analyses.

PET/CT imaging

Tumor volumes and locations were monitored using PET/CT. Animals were injected into their tail veins with 0.4 nmol (10 MBq) in 200 μL of DPBS of $^{11}CH_3$-AS-14 aptamer (six mice) or ^{11}C primer (five mice), and $^{11}CH_3$-unrelated aptamer (four mice) as negative controls or ^{18}F-FDG (two mice) as a positive control. PET/CT analyses with ^{18}F-FDG were performed on the same mice in the day, followed by PET/CT scanning with $^{11}CH_3$-AS-14 aptamer. After 5 min of the procedure, the animals were anesthetized using propofol, a safe anesthesia that does not reduce blood circulation and provides a manipulating window of 15–30 min with a fast recovery. Propofol at an average dose of 75 mg/kg was slowly injected into the mouse's tail until it fell asleep. PET/CT scanning was performed 10, 20, 30, and 40 min after injection. The study was conducted with a Discovery PET/CT 600 scanner (General Electric, United States). It consisted of CT in a spiral mode with 3.75-mm layer thickness followed by post-reconstruction with a 0.625-mm slice. Afterward, positron emission scanning was performed in 3D mode for 5 min with an iterative

reconstruction of the acquired images. Obtained data were analyzed using PET VV software at an AW Volume Share 5 workstation. Radionuclide's accumulation in tumor sites of different localizations, contours, and sizes was estimated. CT images were analyzed using the Hounsfield densitometry scale.

After a 1-day quarantine caused by the necessity to exclude radioactivity, the mice were sacrificed and autopsied to confirm the tumor locations. Tissues were analyzed histologically to prove the presence of cancer cells.

In vivo toxicity study of $^{11}CH_3$-AS-14

Healthy 5-week-old 20-g female ICR mice were injected intramuscularly with the therapeutic dose (0.4 nmol of DNA, 10 MBq) of $^{11}CH_3$-AS-14 in 100 μL of DPBS or pure DPBS (a control group).

The mice were randomly divided into four groups.

Group 1: 20 min after injection of 0.4 nmol (10 MBq) of $^{11}CH_3$-AS-14 in 100 μL of DPBS (five animals per group).

Group 2: 40 min after injection of 0.4 nmol (10 MBq) of $^{11}CH_3$-AS-14 in 100 μL of DPBS (five animals per group).

Group 3: 24 h after injection of 0.4 nmol (10 MBq) of $^{11}CH_3$-AS-14 in 100 μL of DPBS (five animals per group).

Group 3: 24 h after injection of 100 μL of DPBS.

Animals were sacrificed after 20 min (group 1), 40 min (group 2), and 24 h (group 3) of the injection of $^{11}CH_3$-AS-14. The control group was sacrificed 24 h after the injection of DPBS.

After $^{11}CH_3$-AS-14 administration, the radioactivity in the isolated organs was measured using a dosage calibrator (Comecer Petagora, Italy). Organ weight was measured with the analytical balance Ohaus PA-64. Dosage was calculated per gram of the corresponding tissue.

Toxicity was estimated by changes in blood plasma biochemistry (alanine aminotransferase, amylase, alkaline phosphatase, total protein, and bilirubin), performed using a COBAS INTEGRA 400 plus analyzer (Roche Diagnostics, Switzerland). All data were presented as the mean ± standard error of the mean. Hepatotoxicity and nephrotoxicity were estimated by histological analyses of mouse livers and kidneys from different groups.

Tissue analysis

Microscopy analyses of the tumor tissue sections were performed to confirm the presence of tumors in organs. Tissues suspected of containing tumors were harvested and placed in 10% formalin. The tissue sections were fixed onto a glass polylysine slides and stained with hematoxylin-eosin dyes by the standard Blick method. Histological preparations were scanned using a FLASH250 3D HISTECH scanner (Hungary).

Statistical analyses

Statistical data processing was done using the Anaconda Python 3.8 distribution. A two-tailed t test was used to compare group means. Bonferroni correction was applied to p values in the *in vitro* binding of $^{11}CH_3$-AS-14 and the $^{11}CH_3$-unrelated aptamer with the target (Ehrlich) and nontarget cells (hepatocytes).

Five blood serum biochemical parameters in four groups of mice (control, 20 min, 40 min, and 24 h after injection) were compared using ANOVA. A two-tailed t test was used to compare group means. Bonferroni correction was applied to p values.

SUPPLEMENTAL INFORMATION

Supplemental information can be found online at https://doi.org/10.1016/j.omtn.2021.10.020.

ACKNOWLEDGMENTS

This research was performed using equipment of the Federal Siberian Research Clinical Centre under the Federal Medical Biological Agency and Shared Core Facilities of Molecular and Cell Technologies at Krasnoyarsk State Medical University, Krasnoyarsk, Russia. The synchrotron SAXS data were collected at beamline P12 operated by EMBL Hamburg at the PETRA III storage ring (DESY, Hamburg, Germany). We thank Dmitry Molodenskiy for the assistance in using the beamline. The authors are grateful to Mrs. Irina Belyanina, Mrs. Irina Gildenbrant, Mrs. Valentina Grigorieva, Mrs. Daria Tyumentseva, and Dr. Yury Glazyrin for the help with the project realization, and Dr. Fedor V. Arseniev for autopsy guidance. We acknowledge the assistance of Aptamerlab for aptamer characterization and 3D structure analyses. The authors are grateful to Mr. Alexey Kichkailo for the editing of Video S1.

AUTHOR CONTRIBUTIONS

A.V.O. and K.V.B. performed radionuclide synthesis. N.A.T., N.G.C., E.A.K., A.A.V., and O.N.B. performed PET/CT and dosimetry analyses. V.N.S. performed oligonucleotide synthesis. G.S.Z., O.S.K., A.N.B., K.A.L., A.S.K., and T.N.Z. performed cell and animal studies. R.V.M. and V.N.Z. performed SAXS measurements and analyses. N.S., S.V.G., T.E.S., A.S.K., and T.N.Z. performed dosimetry and toxicity analyses. I.A.S. and F.N.T. performed molecular modeling. V.A.K. and S.V.G. performed tissue histological analyses. D.V.V. performed statistical analyses. A.S.K., I.A.S., R.V.M., O.S.K., T.E.S., and D.V.V. made the figures. K.E.A. and S.A.S. analyzed mouse PET/CT scans and prepared mouse scanning protocols. M.V.B., T.N.Z., A.V.O., N.A.T., A.S.K., R.V.M., I.A.S., K.A.L., V.A.K., and S.S.Z. wrote the manuscript. M.V.B., N.G.C., T.N.Z., S.S.Z., and A.S.K. generated the general idea and supervised the project.

DECLARATION OF INTERESTS

Authors declare no competing interests.

REFERENCES

1. Seyfried, T.N., and Huysentruyt, L.C. (2013). On the origin of cancer metastasis. Crit. Rev. Oncog. *18*, 43–73.

2. Wafaie, A.M., Moussa, K.M., and Ebeid, E.M. (2018). Cancer of unknown primary origin: can FDG PET/CT have a role in detecting the site of primary? Egypt. J. Radiol. Nucl. Med. *49*, 190–195.

3. Peck, M., Pollack, H.A., Friesen, A., Muzi, M., Shoner, S.C., Shankland, E.G., Fink, J.R., Armstrong, J.O., Link, J.M., and Krohn, K.A. (2015). Applications of PET imaging with the proliferation marker [18F]-FLT. Q. J. Nucl. Med. Mol. Imaging *59*, 95–104.

4. Bollineni, V.R., Kramer, G.M., Jansma, E.P., Liu, Y., and Oyen, W.J.G. (2016). A systematic review on [18F]FLT-PET uptake as a measure of treatment response in cancer patients. Eur. J. Cancer *55*, 81–97.

5. Positron Emission Tomography: A Guide for Clinicians (2014). (Springer Berlin Heidelberg)

6. Khalid, U., Vi, C., Henri, J., Macdonald, J., Eu, P., Mandarano, G., and Shigdar, S. (2018). Radiolabelled aptamers for theranostic treatment of cancer. Pharmaceuticals (Basel) *12*, 2.

7. Wu, A.M. (2014). Engineered antibodies for molecular imaging of cancer. Methods *65*, 139–147.

8. Warram, J.M., de Boer, E., Sorace, A.G., Chung, T.K., Kim, H., Pleijhuis, R.G., van Dam, G.M., and Rosenthal, E.L. (2014). Antibody-based imaging strategies for cancer. Cancer Metastasis Rev. *33*, 809–822.

9. Bauer, M., Strom, M., Hammond, D.S., and Shigdar, S. (2019). Anything you can do, I can do better: can aptamers replace antibodies in clinical diagnostic applications? Molecules *24*, 4377.

10. Marx, V. (2013). Calling the next generation of affinity reagents. Nat. Methods *10*, 829–833.

11. Tuerk, C., and Gold, L. (1990). Systematic evolution of ligands by exponential enrichment: RNA ligands to bacteriophage T4 DNA polymerase. Science *249*, 505–510.

12. Ellington, A.D., and Szostak, J.W. (1990). In vitro selection of RNA molecules that bind specific ligands. Nature *346*, 818–822.

13. Gijs, M., Aerts, A., Impens, N., Baatout, S., and Luxen, A. (2016). Aptamers as radiopharmaceuticals for nuclear imaging and therapy. Nucl. Med. Biol. *43*, 253–271.

14. Missailidis, S., and Perkins, A. (2007). *Update:* aptamers as novel radiopharmaceuticals: their applications and future prospects in diagnosis and therapy. Cancer Biother. Radiopharm. *22*, 453–468.

15. Khalid, U., Vi, C., Henri, J., Macdonald, J., Eu, P., Mandarano, G., and Shigdar, S. (2018). Radiolabelled aptamers for theranostic treatment of cancer. Pharmaceuticals *12*, 2.

16. Yoon, S., and Rossi, J.J. (2018). Targeted molecular imaging using aptamers in cancer. Pharmaceuticals (Basel) *11*, 71.

17. Kryza, D., Debordeaux, F., Azéma, L., Hassan, A., Paurelle, O., Schulz, J., Savona-Baron, C., Charignon, E., Bonazza, P., Taleb, J., et al. (2016). Ex vivo and in vivo imaging and biodistribution of aptamers targeting the human matrix MetalloProtease-9 in melanomas. PLoS One *11*, e0149387.

18. Li, J., Zheng, H., Bates, P.J., Malik, T., Li, X.-F., Trent, J.O., and Ng, C.K. (2014). Aptamer imaging with Cu-64 labeled AS1411: preliminary assessment in lung cancer. Nucl. Med. Biol. *41*, 179–185.

19. Zhu, G., Zhang, H., Jacobson, O., Wang, Z., Chen, H., Yang, X., Niu, G., and Chen, X. (2017). Combinatorial screening of DNA aptamers for molecular imaging of HER2 in cancer. Bioconjug. Chem. *28*, 1068–1075.

20. Kolovskaya, O.S., Zamay, T.N., Zamay, A.S., Glazyrin, Y.E., Spivak, E.A., Zubkova, O.A., Kadkina, A.V., Erkaev, E.N., Zamay, G.S., Savitskaya, A.G., et al. (2014). DNA-aptamer/protein interaction as a cause of apoptosis and arrest of proliferation in Ehrlich ascites adenocarcinoma cells. Biochem. Mosc. Suppl. Ser. Membr. Cell Biol. *8*, 60–72.

21. Belyanina, I.V., Zamay, T.N., Zamay, G.S., Zamay, S.S., Kolovskaya, O.S., Ivanchenko, T.I., Denisenko, V.V., Kirichenko, A.K., Glazyrin, Y.E., Garanzha, I.V., et al. (2017). *In vivo* cancer cells elimination guided by aptamer-functionalized Gold-coated magnetic nanoparticles and controlled with low frequency alternating magnetic field. Theranostics *7*, 3326–3337.

22. Forte, E., Fiorenza, D., Torino, E., Costagliola di Polidoro, A., Cavaliere, C., Netti, P.A., Salvatore, M., and Aiello, M. (2019). Radiolabeled PET/MRI nanoparticles for tumor imaging. J. Clin. Med. *9*, 89.

23. van Dongen, G.A.M.S., Visser, G.W.M., Lub-de Hooge, M.N., de Vries, E.G., and Perk, L.R. (2007). Immuno-PET: a navigator in monoclonal antibody development and applications. Oncologist *12*, 1379–1389.

24. van Dongen, G.A.M.S., Poot, A.J., and Vugts, D.J. (2012). PET imaging with radiolabeled antibodies and tyrosine kinase inhibitors: immuno-PET and TKI-PET. Tumor Biol. *33*, 607–615.

25. Niu, G., Lang, L., Kiesewetter, D.O., Ma, Y., Sun, Z., Guo, N., Guo, J., Wu, C., and Chen, X. (2014). In vivo labeling of serum albumin for PET. J. Nucl. Med. *55*, 1150–1156.

26. Thorneloe, K.S., Sepp, A., Zhang, S., Galinanes-Garcia, L., Galette, P., Al-Azzam, W., Vugts, D.J., van Dongen, G., Elsinga, P., Wiegers, J., et al. (2019). The biodistribution and clearance of AlbudAb, a novel biopharmaceutical medicine platform, assessed via PET imaging in humans. EJNMMI Res. *9*, 45.

27. Goldberg, H.L., Moses, J.W., Borer, J.S., Fisher, J., Tamari, I., Skelly, N.T., and Cohen, B. (1983). Exercise left ventriculography utilizing intravenous digital angiography. J. Am. Coll. Cardiol. *2*, 1092–1098.

28. Sun, X., Li, Y., Liu, T., Li, Z., Zhang, X., and Chen, X. (2017). Peptide-based imaging agents for cancer detection. Adv. Drug Deliv. Rev. 110–111.

29. Graewert, M.A., Da Vela, S., Gräwert, T.W., Molodenskiy, D.S., Blanchet, C.E., Svergun, D.I., and Jeffries, C.M. (2020). Adding size exclusion chromatography (SEC) and light scattering (LS) devices to obtain high-quality small angle X-ray scattering (SAXS) data. Crystals *10*, 975.

30. Mertens, H.D.T., and Svergun, D.I. (2010). Structural characterization of proteins and complexes using small-angle X-ray solution scattering. J. Struct. Biol. *172*, 128–141.

31. Trewhella, J., Duff, A.P., Durand, D., Gabel, F., Guss, J.M., Hendrickson, W.A., Hura, G.L., Jacques, D.A., Kirby, N.M., Kwan, A.H., et al. (2017). 2017 publication guidelines for structural modelling of small-angle scattering data from biomolecules in solution: an update. Acta Crystallogr. Sect. Struct. Biol. *73*, 710–728.

32. Konarev, P.V., Volkov, V.V., Sokolova, A.V., Koch, M.H.J., and Svergun, D.I. (2003). *Primus* : a windows PC-based system for small-angle scattering data analysis. J. Appl. Crystallogr. *36*, 1277–1282.

33. Franke, D., Petoukhov, M.V., Konarev, P.V., Panjkovich, A., Tuukkanen, A., Mertens, H.D.T., Kikhney, A.G., Hajizadeh, N.R., Franklin, J.M., Jeffries, C.M., et al. (2017). *Atsas 2.8*: a comprehensive data analysis suite for small-angle scattering from macromolecular solutions. J. Appl. Crystallogr. *50*, 1212–1225.

34. Semenyuk, A.V., and Svergun, D.I. (1991). Gnom – a program package for small-angle scattering data processing. J. Appl. Crystallogr. *24*, 537–540.

35. Svergun, D.I. (1999). Restoring low resolution structure of biological macromolecules from solution scattering using simulated annealing. Biophys. J. *76*, 2879–2886.

36. Owczarzy, R., Tataurov, A.V., Wu, Y., Manthey, J.A., McQuisten, K.A., Almabrazi, H.G., Pedersen, K.F., Lin, Y., Garretson, J., McEntaggart, N.O., et al. (2008). IDT SciTools: a suite for analysis and design of nucleic acid oligomers. Nucleic Acids Res. *36*, W163–W169.

37. Hanwell, M.D., Curtis, D.E., Lonie, D.C., Vandermeersch, T., Zurek, E., and Hutchison, G.R. (2012). Avogadro: an advanced semantic chemical editor, visualization, and analysis platform. J. Cheminform. *4*, 17.

38. Fedorov, D.G., and Kitaura, K. (2007). Pair interaction energy decomposition analysis. J. Comput. Chem. *28*, 222–237.

39. Gaus, M., Lu, X., Elstner, M., and Cui, Q. (2014). Parameterization of DFTB3/3OB for sulfur and phosphorus for chemical and biological applications. J. Chem. Theor. Comput. *10*, 1518–1537.

40. Nishimoto, Y., and Fedorov, D.G. (2016). The fragment molecular orbital method combined with density-functional tight-binding and the polarizable continuum model. Phys. Chem. Chem. Phys. *18*, 22047–22061.

41. Schmidt, M.W., Baldridge, K.K., Boatz, J.A., Elbert, S.T., Gordon, M.S., Jensen, J.H., Koseki, S., Matsunaga, N., Nguyen, K.A., Su, S., et al. (1993). General atomic and molecular electronic structure system. J. Comput. Chem. *14*, 1347–1363.

42. Svergun, D., Barberato, C., and Koch, M.H.J. (1995). *Crysol* – a program to evaluate X-ray solution scattering of biological macromolecules from atomic coordinates. J. Appl. Crystallogr. *28*, 768–773.

43. Panjkovich, A., and Svergun, D.I. (2016). SASpy: a PyMOL plugin for manipulation and refinement of hybrid models against small angle X-ray scattering data. Bioinformatics *32*, 2062–2064.

44. DeLano, W.L. (2002). Pymol: An Open-Source Molecular Graphics Tool.*40* (CCP4 Newslett. on Protein crystallogr.), pp. 82–92.

OMTN, Volume *26*

Supplemental information

^{11}C-radiolabeled aptamer for imaging of tumors and metastases using positron emission tomography- computed tomography

Anastasia V. Ozerskaya, Tatiana N. Zamay, Olga S. Kolovskaya, Nikolay A. Tokarev, Kirill V. Belugin, Natalia G. Chanchikova, Oleg N. Badmaev, Galina S. Zamay, Irina A. Shchugoreva, Roman V. Moryachkov, Vladimir N. Zabluda, Vladimir A. Khorzhevskii, Nikolay Shepelevich, Stanislav V. Gappoev, Elena A. Karlova, Anastasia S. Saveleva, Alexander A. Volzhentsev, Anna N. Blagodatova, Kirill A. Lukyanenko, Dmitry V. Veprintsev, Tatyana E. Smolyarova, Felix N. Tomilin, Sergey S. Zamay, Vladimir N. Silnikov, Maxim V. Berezovski, and Anna S. Kichkailo

^{11}C-Radiolabeled Aptamer For Imaging of Tumor and Metastasis Using Positron Emission Tomography - Computed Tomography

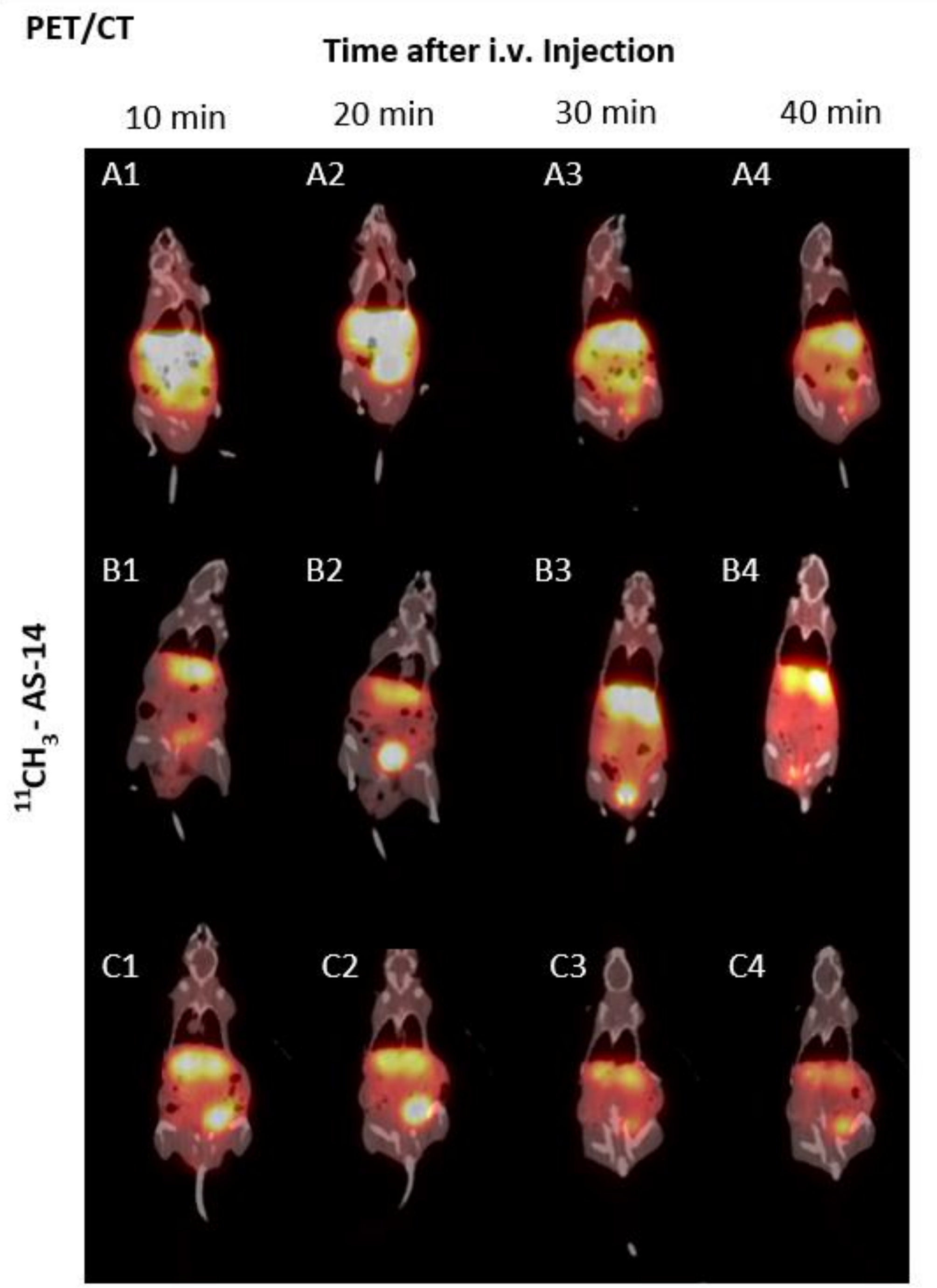

Figure S1. PET/CT imaging of $^{11}CH_3$-AS-14 of three mice with Ehrlich's tumor metastases. The scans were performed at 10, 20, 30, and 40 minutes after tail vein injection of 0.4 nmol $^{11}CH_3$-AS-14 in 200 µl of DPBS.

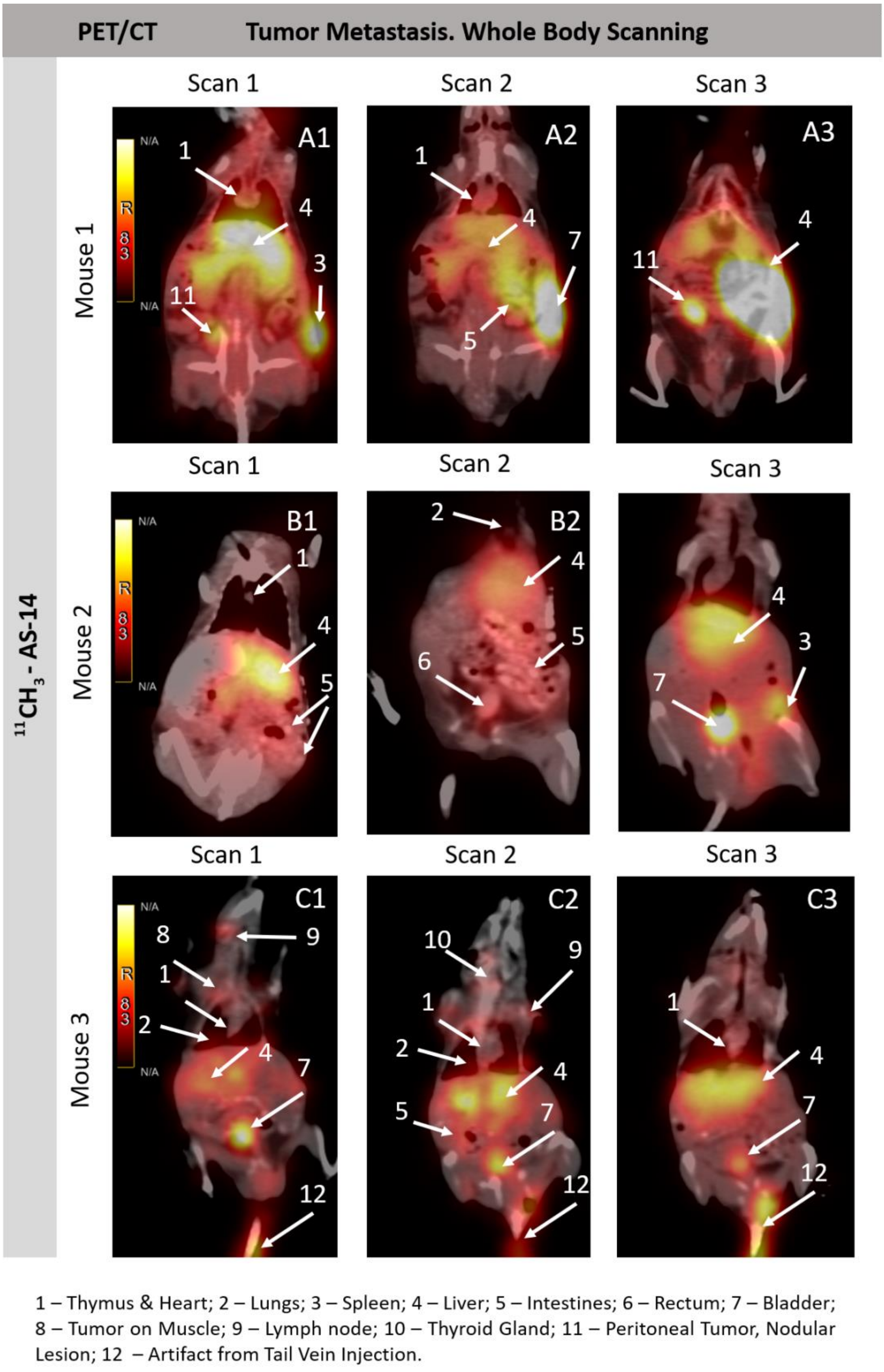

Figure S2. Whole body PET/CT scanning after injection of $^{11}CH_3$-AS-14 probe reveals tumor localization in different organs. The PET/CT images of 3 representative mice with metastases (3 scans per mouse). Arrows indicate the tumor sites in different organs.

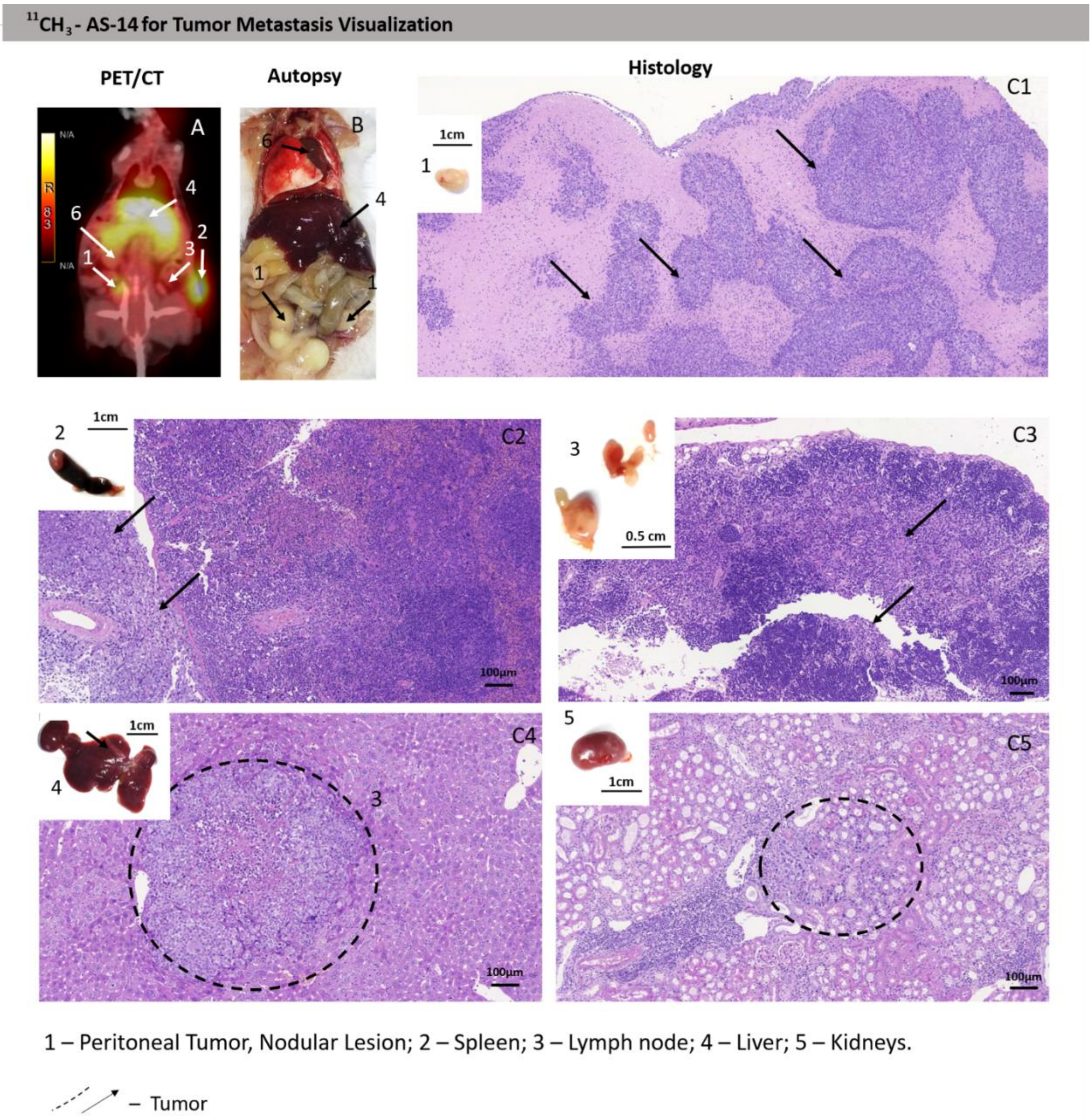

Figure S3. Tumor and metastases localization using the $^{11}CH_3$-AS-14 probe and PET/CT. The representative PET/CT images of mice with metastases (A) were confirmed by autopsy (B) and histological analyses of metastatic tissues (C): peritoneal tumor, nodular lesion (C1), spleen (C2), lymph node (C3), liver (C4), and kidneys (C5). Hematoxylin & Eosin, staining. Magnification C1 – ×30; C2,5 - ×100. Arrows and dashed circles indicate the tumor sites in different organs at PET/CT, autopsy, and correspondent tissue sections.

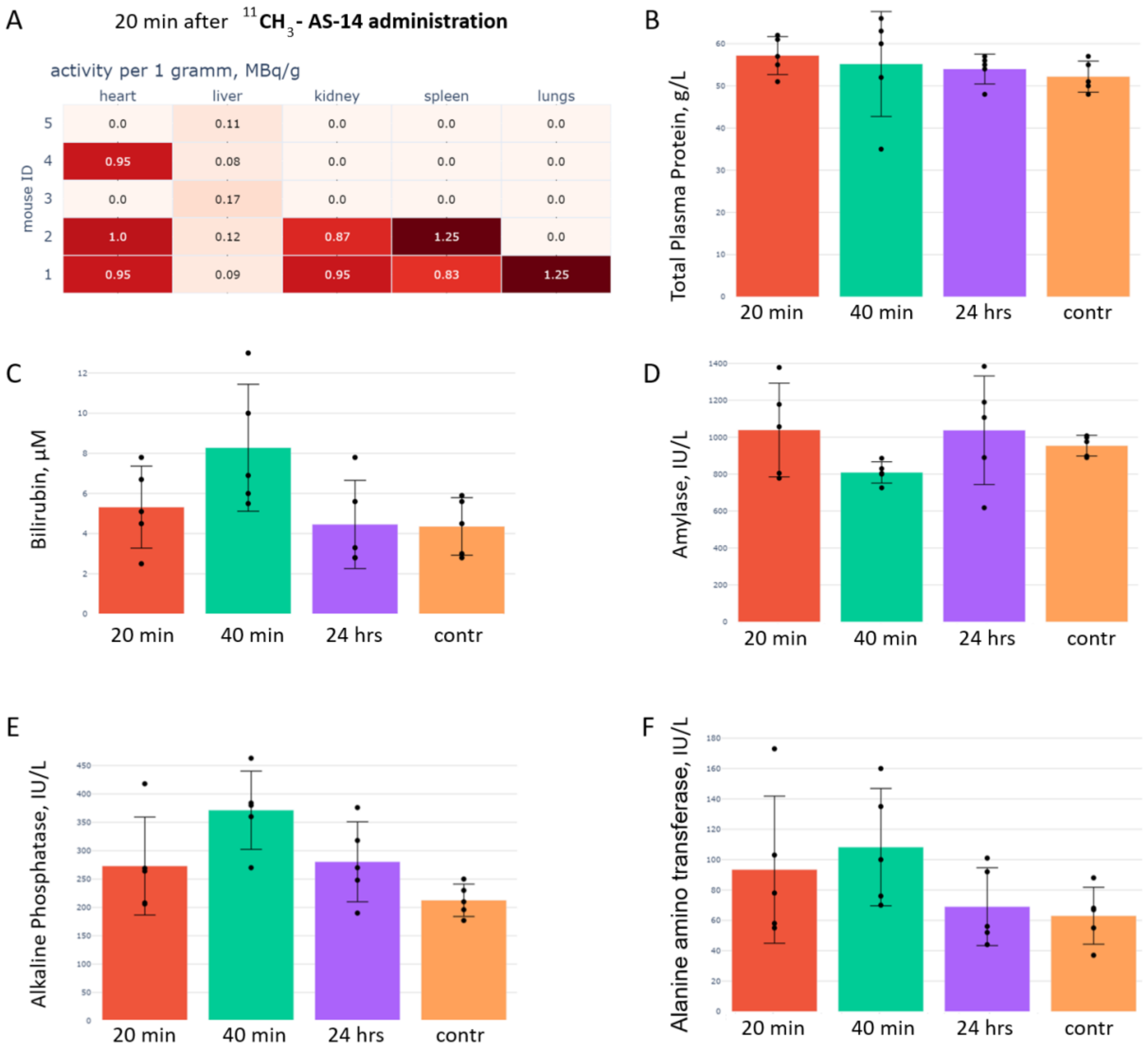

Figure S4. Acute toxicity study of $^{11}CH_3$-AS-14. Accumulation of radioactivity in different mouse organs after 20 minutes of intramuscular $^{11}CH_3$-AS-14 administration (A). Blood serum biochemistry after 20, 40 minutes and 24 hours of intramuscular $^{11}CH_3$-AS-14 administration: levels of total plasma protein (B) and bilirubin (C), activities of amylase (D), alkaline phosphatase (E) and alanine aminotransferase (F).

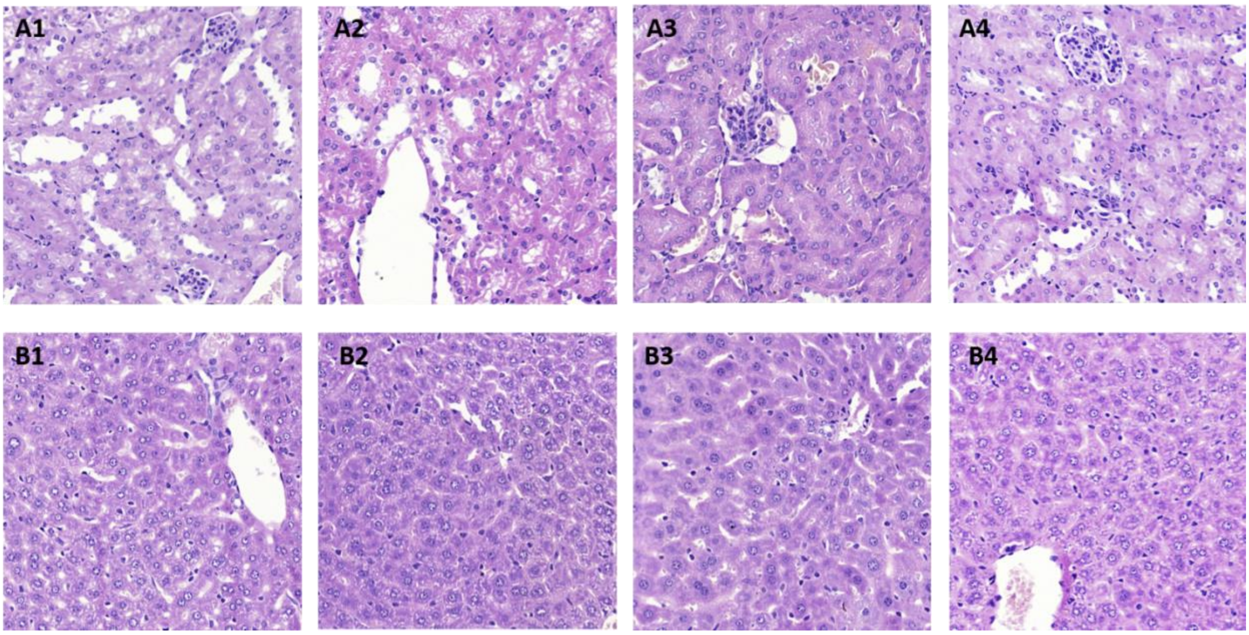

Figure S5. Histological examinations of mice kidneys and livers after intramuscular $^{11}CH_3$-AS-14 administration. Kidneys (A) and livers (B) after 20 (1), 40 (2) minutes and 24 hours (3) comparing with a DPBS-injected control group (4).

Supplemental Video. Comparative PET/CT imaging using $^{11}CH_3$-AS-14 with ^{18}F-FDG.
